# Modified Harris Hawks optimization for the 3E feasibility assessment of a hybrid renewable energy system

**DOI:** 10.1038/s41598-024-70663-5

**Published:** 2024-08-29

**Authors:** Asmita Ajay Rathod, Balaji S

**Affiliations:** School of Electrical Engineering, Vellore Institute of Technology, Vellore, Tamil Nadu India

**Keywords:** Hybrid Energy System, Optimal Sizing, Modified Harris Hawks Optimization, Cost of Energy, 3E Analysis, Environmental sciences, Energy science and technology, Engineering, Mathematics and computing

## Abstract

The off-grid Hybrid Renewable Energy Systems (HRES) demonstrate great potential to be sustainable and economically feasible options to meet the growing energy needs and counter the depletion of conventional energy sources. Therefore, it is crucial to optimize the size of HRES components to assess system cost and dependability. This paper presents the optimal sizing of HRES to provide a very cost-effective and efficient solution for supplying power to a rural region. This study develops a PV-Wind-Battery-DG system with an objective of 3E analysis which includes Energy, Economic, and Environmental CO_2_ emissions. Indispensable parameters like technical parameters (Loss of Power Supply Probability, Renewable factor, PV fraction, and Wind fraction) and social factor (Human Developing Index) are evaluated to show the proposed modified Harris Hawks Optimization (mHHO) algorithm’s merits over the existing algorithms. To achieve the objectives, the proposed mHHO algorithm uses nine distinct operators to obtain simultaneous optimization. Furthermore, the performance of mHHO is evaluated by using the CEC 2019 test suite and the most optimal mHHO is chosen for sizing and 3E analysis of HRES. The findings demonstrate that the mHHO has achieved optimized values for Cost of Energy (COE), Net Present Cost (NPC), and Annualized System Cost (ASC) with the lowest values being 0.14130 $/kWh, 1,649,900$, and 1,16,090$/year respectively. The reduction in COE value using the proposed mHHO approach is 0.49% in comparison with most of the other MH-algorithms. Additionally, the system primarily relies on renewable sources, with diesel usage accounting for only 0.03% of power generation. Overall, this study effectively addresses the challenge of performing a 3E analysis with mHHO algorithm which exhibits excellent convergence and is capable of producing high-quality outcomes in the design of HRES. The mHHO algorithm attains optimal economic efficiency while simultaneously minimizing the impact on the environment and maintaining a high human development index.

## Introduction

Electricity does not exclusively indicate energy. Energy is a crucial foundation of the economy as well as a feasible means of promoting sustainable growth. In order to achieve continuous economic and social progress, it is essential to have sufficient and continuous access to electricity^1^. However, approximately 1.2 billion individuals constituting 17% of the global population have no access to electricity which is primarily in rural areas^2^. Rural regions are typically situated at a considerable distance from the national power grid and are often situated in challenging terrain such as mountainous regions or dense forests. The extension of transmission lines can be prohibitively expensive or impractical in these remote areas. The economic growth of the country is significantly influenced by electricity. Expanding economies such as India require a greater energy supply than what is currently available to adequately address their rising energy demands. The burgeoning energy demands are primarily due to the technology advancements and other enhancements in each stage of mankind. In other words, the countries have an energy shortage and they need cost-effective methods to address it. The issue of energy in the global context remains a challenge due to the need for diversification regardless of the advancements made in the exploitation of sustainable energy resources.

**Fig. 1:**
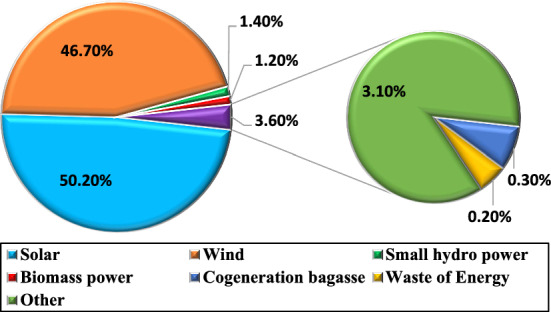
Different RES contributions in India.

At present there is a growing global inclination towards the advancement of Renewable Energy Sources (RESs). Consequently, there has been an expanding imperative to cultivate sustainable and eco-friendly energy sources commonly referred to as RESs. Solar, wind, small hydro, cogeneration bagasse, and biomass, are just a few of the sources of renewable energy that have great potential to be used to power developing economies. For example on March 31, 2021, it was predicted that India has a total renewable power generating capacity of 14,90,727 MW^3^ and the full overview of the contributions provided by different RES in India is shown in Fig. 1.

Each specific RES exhibits a unique set of advantages and disadvantages. Solar and wind energy as sources of renewable energy have become known for their cleanliness, affordability, and environmental sustainability. It is important to note that their effectiveness is contingent upon various factors such as weather conditions, climatic patterns, and specific geographical regions. Despite the intermittent and unpredictable nature of solar and wind sources, which can be attributed to abrupt changes in solar radiation as well as the speed of the wind, they can serve as viable substitutes for fossil fuels in power generation^4^. Due to their unpredictable nature, RESs that rely on a single source, like solar and wind power, are unable to provide the year-round need of electricity. However, the hybridization of solar Photovoltaic (PV) and Wind Turbine (WT) technologies have the potential to fulfill demand for electricity under the condition of adequate solar irradiation and wind speeds. As said already, the solar and wind sources are intermittent, thus there exists a requirement for an Energy Storage System (ESS). Numerous technologies for storing energy have been implemented in various applications, encompassing batteries, hydrogen, compressed air, flywheels, pumped hydro storage, and gravity energy. Batteries are extensively utilized as a short-term storage medium owing to their affordability, ease of upkeep or maintenance, and consistent operational capabilities. Green hydrogen possesses several notable benefits including its ability to store energy over extended periods, and its capacity to mitigate local pollutants in an environmentally friendly manner. Also, the specialty of Green Hydrogen is the complete absence of CO_2_ emissions. However, it is important to acknowledge that green hydrogen is accompanied by a comparatively higher cost. At present, the water electrolysis process stands as the most ecologically sustainable method for hydrogen production with a drawback of substantial electricity consumption^5^. The utilization of hydrogen fuel cells facilitates the conversion of the energy stored within hydrogen into electrical energy.

It has become increasingly clear that a Hybrid Energy System (HES) that incorporates mixed generation and suitable storage is necessary to meet the requirements for a consistent, sustainable, and dependable power supply. As a result, determining the optimal sizing for these systems has become a highly desired research topic worldwide. By implementing appropriate component sizing techniques and adopting an effective power management strategy or energy dispatch strategy, it is possible to establish a Hybrid Renewable Energy System (HRES) that offers a sustainable, economically viable, and dependable solution for fulfilling the energy requirements of developing nations. A key consideration in the design of HRES is the sizing of its components. Solar and Wind energy sources exhibit discontinuity, dependence on specific locations, and significant variability^6^. Consequently, the economic viability of stand-alone energy systems is compromised due to the inability to properly match power generation with energy demand fluctuations^7^. HRES which utilize solar PV, wind technologies, ESSs, and Diesel Generators (DGs) have the potential to serve as viable energy sources^8^.

The optimization and efficient operation of HRES will contribute to the enhanced dependability, reliability, and control, along with the efficiency of the power network. Efficient management of these components is crucial for enhancing the HRES operation or performance. The implementation strategy of HRES is determined by technical, economic, and environmental indicators within the network^9^. In recent times, there has been a significant focus on the identification of the most suitable HRES configuration and capacity. The HRES is established purely by taking into consideration the presence of RESs and annual power demands specific to a given location. The determination of a suitable size for the HRES through the application of techno-economic evaluation has been a prominent area of research for the past quarter-century or over 25 years.

Literature explores the optimum size of off-grid systems to reduce the Net Present Cost (NPC), Cost of Energy (COE) or Levelized Cost of Energy (LCOE), and Loss of Power Supply Probability (LPSP) while maximizing the renewable factor (RF)^10^. SK.A. Shezan et al. performed a practical evaluation of an independent WT/PV/DG/battery HES designed for rural areas. The HOMER software tool was employed to model and enhance the efficiency of the PV/WT/DG hybrid energy system using real-time field data of solar radiation as well as wind speed pertaining to the given area. The simulations using HOMER software usually confirms that the framework is feasible in relation to NPC analysis and the idea of decreasing CO_2_ emissions^11^. The various metrics including NPV (Net Present Value), energy expenditure, energy production, ASC, EE (Energy Efficiency) created, and pollution produced can be computed using HOMER. The analysis demonstrated that the combination of PV/DG sets results in the lowest NPC and COE^12^. Laura Tribioli and Raffaello Cozzolino performed a Techno-economic assessment of an autonomous microgrid (MG) for a commercial building in eight different temperature zones. A comparative study is performed to assess the technical, economic, and environmental implications using Simulink and HOMER for the grid^13^. A study was conducted by Ashkan Toopshekan et al. on the integration of PV, WT, DG, and BES systems for residential areas. The study was focused on developing a new dispatch method that resulted in improved COE and reliability factors^14^. As reported by many researchers if RES penetration rises in a given region, pollutant gas emissions are minimized^15^.

Anis Afzal, Mohibullah Mohibullah, and Virendra Kumar Sharma performed comparative research on optimal HRES to provide energy security and their work focused on the sizing, generator operating hours, and sensitivity analysis^16^. Rahman Tito et al. carried out an investigation on optimizing the size of wind-photovoltaic hybrid energy systems to more effectively manage transient load conditions^17^. Ramin Hosseinalizadeh et al. developed an optimization-simulation model to economically determine the size of an HRES consisting of PV, WT, and fuel cell (FC) technologies. The system is designed for stand-alone applications^18^. Om Krishan and Sathans Suhag presented a techno-economic study and optimal design of an HRES. It was designed to fulfill the electricity needs of a community in Yamunanagar district, Haryana, India, which currently lacks access to sufficient energy for residential and agricultural purposes^19^. Shafiqur Rehman et al. conducted a techno-economic study that included optimizing the size and implementing Model Predictive Control of Standalone HRES components. The goal was to fulfill the residential load demand of a specified region^20^. Sanjay et al. conducted a techno-commercial evaluation of HES for agriculture farms utilizing HOMER Software^21^. Shen Yuong Wong and Chunze Li conducted a techno-economic study of the most efficient HRES for an MG on a campus^22^. The study by Joshi Sukhdev Nirbheram, Aeidapu Mahesh, and Ambati Bhimaraju examined the best HRES size based on source degradation. Additionally, the number of BESS to be included in HRES to fulfill the given restrictions and their influence on energy costs was analyzed^23^.

Also, Elkadeem et al. proposed a design and control structure for large-scale PV/WTG hybrid systems with and without the integration of BES. Based on the proposed eight cases by Elkadeem et al., it is found that the grid-connected WT/PV/converter system case was the most cost-effective and ecological one. Control enhances renewable power monitoring as well as reliability. It provides a framework for large-scale renewable energy initiatives^24^. Mersad Shoaei, Ahmad Hajinezhad, and Seyed Farhan Moosavian developed an innovative hybrid power system that integrates gas, steam, and organic Rankine cycles with RES sources such as geothermal and solar. The system's performance was analyzed, and appropriate fluids for the organic cycles were identified. These fluids enhance the efficiency, power production, and lower the emissions in comparison to a conventional system^25^. Dawoud et al. explored the impact of implementing demand-side management (DSM) techniques on MGs located in distant places, with a focus on optimizing energy consumption. They presented a new approach to construct MGs that taken into account technological, environmental, and economic considerations. The findings demonstrated that DSM has the capacity to mitigate peak energy demand, minimize expenses, and diminish greenhouse gas emissions, hence enhancing the sustainability and adaptability of MGs^26^. Rahimi-Esbo et al., examined innovative approaches to develop off-grid HRES for telecommunications towers. The design proposed by them includes solar panels, hydrogen technology, as well as an organic Rankine cycle to enhance energy efficiency, recover waste heat, and minimize expenses. The analysis of the design determined optimal configurations for these elements, resulting in superior effectiveness, minimal expenses, and less ecological footprint^27^. Andrey Nikitin et al., examined a multi-generation system that utilizes solar and wind power to provide electricity for near-zero energy buildings. The multi-generation system simulation modeled the year-round performance of four Russian towns, considering various factors such as energy consumption, efficiency, cost, and environmental effects. The system offers cooling, heating, and electric power generation, along with freshwater production, demonstrating exceptional efficiency and a substantial decrease in CO_2_ emissions when compared to conventional systems^28^. Various techniques have been reported in the literature to analyze and enhance the performance of HRES. Table 1 provides a summary of the prior optimization works in the optimal design of HRES components.

**Table 1:** An overview of relevant research on optimizing the performance of HRES.

Year	Configuration						Load Type	Software/optimization method	Objective function	Pros/cons	References
	PV	WTG	BES	DG	HESS	BMG					
2021	✔	** × **	** × **	✔	✔	** × **	Institute load	HOMER	LCOE	The employed tool HOMER is a very efficient tool for determining the appropriate sizes of hybrid renewable energy storage systems. But it is subject to certain constraints. For instance, it does not optimize or design a multi-objective problem, nor does it allow intra-hour variability with depth of drain of the battery. In addition, it also requires a significant amount of processing time for bigger design problems.	^29^
2021	✔	✔	✔	** × **	** × **	** × **	Rural area	HOMER	LCC	Examined many strategies for sizing and optimizing wind turbines and photovoltaic systems to meet peak energy demand within the constraints of available sources. But, only Life Cycle Cost (LCC) was determined for PV/WT/BES configuration.	^30^
2021	✔	✔	✔	✔	×	×	Residential load	Recursive algorithm	NPC, COE	The study examined the size of two configurations for MG systems: (1) a PV/BES, and (2) a PV/WT/BES by considering the autonomous days. However, effective iterative energy management using recursive algorithms used was difficult to converge, particularly for complex systems. This may raise the computational burden and longer response times	^31^
2022	✔	✔	✔	✔	** × **	** × **	Household and the reverse osmosis system, (City of Bernice, Egypt)	Multi-objective multi-verse optimization (MOMVO) algorithm	COE	Different configurations were modeled and optimized. However, MOMVO can be computationally expensive because it involves a complex optimization process to find the best solution across multiple objectives. Finding the ideal balance between these objectives can be challenging with MOMVO	^32^
2022	✔	✔	✔	** × **	** × **	** × **	500 households with 500 EVs, Kanyakumari, India	Improved search space reduction algorithm	LCOE	Optimizing an HRES with EVs was considered. However, it only included limited EVs and doesn't account for their unexpected charging patterns on long-term system dependability and cost. Also, it does not conduct a comprehensive comparison of its optimization algorithm (SSR) with established methods, apart from a basic benchmark	^33^
2023	✔	✔	✔	×	×	×	Residential Load	JGWO	ASC	The economic study was carried out for 5 different LPSP values. However, JGWO can suffer from increased complexity because it required to tune parameters from both algorithms	^34^
2023	✔	✔	✔	✔	** × **	** × **	Rural area, Uttarakhand (India)	Moth flam optimization (MFO)	NPC, COE	Focussed on the economic aspects of an HRES for electrification but doesn't considered the variability of renewables or broader impacts like social and environmental factors	^35^
2023	✔	✔	✔	✔	×	×	Village, Malaysia	Grey Wolf Optimizer (GWO)	NPC, COE	The GWO method for optimum size may involve tuning many parameters, increasing complexity, and also being stuck on local optima while searching for the ideal design. This may result in an economically non-ideal configuration for the MG	^36^
2024	✔	✔	✔	✔	×	×	Shopping complex (SC) and EVCS, university campus, India	Ebola Optimization Search Algorithm (ESOA)	NPC, LCOE	Real-world dynamics was oversimplified by the presumptions of continuous grid capabilities and a homogenous load profile for electric cars. The work was depended on limited meteorological data which resulted in poor estimate of renewable sources and adoption of optimization methodologies.	^37^
2024	✔	✔	✔	** × **	** × **	✔	Rural community, Uttarakhand, India	Giza pyramids construction (GPC)	ASC, LCOE	Proposed a method (GPC) to optimize a hybrid solar-wind-battery-biomass system for rural electrification. While GPC outperforms another method (PSO) in cost and speed, the system itself doesn't consider factors like intermittency of renewables or social/environmental impacts	^38^
2024	✔	✔	✔	×	×	×	Airport cargo terminal	Triangle evaluation scheme	LCOE	Provided a new renewable MG evaluation approach, however it has only been tested at one airport cargo terminal. The greater use of load shedding for flexibility may not be suited for all applications. One of the limitations of the specific method employed was its failure to consider the broader economic or social consequences of increased load shedding	^39^
2024	✔	** × **	** × **	** × **	** × **	** × **	Seawater reverse osmosis (SWRO) plant, Northwestern Egypt	HOMER	LCOE, levelized cost of water (LCOW)	Investigated the design and performance of a desalination system that was powered by solar energy. Although the model identifies substantial economic and environmental advantages, its long-term efficacy cannot be confirmed due to the absence of real-world implementation data. Furthermore, it relies on HOMER software, which might limit the complexity of real-world system modeling	^40^

The application of Meta-heuristic (MH) algorithms have been observed in addressing the challenge of sizing and performing techno-economic analysis for grid-connected or off-grid HRES^41^. Over the past two decades, several MH optimization algorithms have been developed and inspired by various natural phenomena. The Artificial Bee Colony (ABC) algorithm draws inspiration from the remarkable behavior of honey bee swarms^42^ and the Grey Wolf Optimizer (GWO) motivated by the grey wolves^43^. Likewise, the Dolphin Echolocation (DE) optimization algorithm was inspired by the hunting strategies utilized by dolphins^44^ and the Elephant Search Algorithm (ESA) is modeled on the behavioral traits of the elephant herd^45^. One of the conventional algorithms like Whale Optimization Algorithm (WOA) is inspired by the adoption of bubble nets with humpback whales as a hunting technique^46^. Other algorithms of interest like the Spotted Hyena Optimizer (SHO) encouraged by spotted hyenas' behavior^47^, and the Red Fox Optimization algorithm (RFO) is motivated by the concept of tracking and managing the population of a well-known species the red fox^48^, and so on. However, it is essential to note that these algorithms demonstrate the inherent capabilities to address the challenges dependent on the complexity involved. According to the No Free Lunch (NFL) theorem not all MH algorithms can handle all types of optimization problems^49^. Hence problem-based improvements or modifications are required to enhance their performance. Each MH algorithm has advantages and disadvantages of its own.

Moreover, it is essential to note that these MH algorithms exhibit various degrees of accuracy as well as effectiveness while solving a specific challenge. In recent years, various types of new and highly efficient MH algorithms have been developed and extensively utilized to solve complex and non-linear optimization challenges. Further, sizing and techno-economic analysis for HRES challenges transformed into an optimization challenge. MH optimization can solve these challenges such as minimizing Net Present Cost (NPC), Annualized System Cost (ASC), Cost of Energy (COE), Loss of Power Supply Probability (LPSP), improved Greenhouse Gas Emission (GHGE) savings, Human Development Index (HDI) and maximum reliability of the system. The Harris Hawks Optimization (HHO) algorithm proposed by Ali Asghar Heidari et al.^50^ has attracted attention in the field of optimization challenges.

According to the literature, the HHO algorithm is applied to the sizing, optimization, and design of autonomous MG, and various applications have been proposed recently^51^. Further renewable energy-based DG unit sizing and planning for the IEEE bus system are also analyzed^52^. Gauri Sahoo et al. presented an approach including the utilization of Modified HHO to develop a Fractional-Order Fuzzy Proportional-Integral-Derivative (PID) controller. The controller was aimed to regulate the frequency of a multi-MG system^53^. Mohammed Kharrich et al. developed an equilibrium optimized for the techno-economic analysis of MGs and the results are compared with the HHO algorithm^54^. Ch Srivardhan Kumar et al. provided a control approach that offers a reduced level of complexity for voltage compensation to boost power quality inside the distribution systems. The control technique proposed by them utilized a customized power device known as the Dynamic Voltage Restorer (DVR), which was implemented with various inverter topologies with the application of the HHO algorithm^55^. Zhang et al. proposed a method for phase partitioning using HHO with a hard sequential constraint. It was proposed to find the optimal phase partitioning results within a specific target phase number in the inner loop. Additionally, it automatically finds the optimal phase number in the outer loop by balancing the trade-off between modeling complexity as well as partition execution^56^.

The fundamental concept of this HHO technique relies on the hunting strategy employed by the Harries Hawk in capturing its prey. The HHO algorithm is a relatively new and highly promising optimization methodology for several reasons. The HHO theory is based on a population approach and does not rely on the computation of costly partial derivatives. As a result, it is classified as a gradient or derivative-free optimization method^57^. The aforementioned benefit enables the utilization of HHO in the optimization of any problem, provided that the problem is well formulated. It is recognized as a global optimization algorithm due to its incorporation of both exploitation as well as exploration phases. However, the optimal convergence curve as well as local minima are two challenges reported in the literature. To mitigate this challenge a modified version of the HHO (m-HHO) algorithm is proposed in this work.

The research proposed in this work utilizes a mHHO method to identify the optimal size of the HRES system. This is the first and innovative approach where HHO is modified with nine different operators using inertia weight strategies. The effectiveness of these improved algorithms has been tested by utilizing CEC 2019 test functions^58^. In addition, the statistical importance of this algorithm is determined by using a statistical test known as the Friedman rank (f-rank) test^59^. This statistical test is non-parametric in nature. The f-rank test involves assigning a distinct rank to each algorithm being compared based on its performance. The total f-rank is then determined by considering the results across all test functions. Box plots and statistical test studies have also been performed to check the accuracy as well as the robustness of the proposed algorithm. From the acquired results best mHHO is selected for HRES design and the results of the design are compared with some well-known MH-algorithms such as Differential Evolution (DE) algorithm^60^, Enhanced Whale Optimization Algorithm (EWOA)^61^, Particle Swarm Optimization (PSO)^62^, jDE100^63^, Novel Bat Algorithm (NBA)^64^ and Sin Cosine Algorithm (SCA)^65^. Also, the f-rank test and Wilcoxon test (p-rank) are carried out to verify the effectiveness and robustness of the mHHO algorithm for picking the optimal mHHO to design the hybrid system.

From the literature, it is noted that the environmental, economic, and ecological (3E) assessment along with social and technological elements on optimal sizing of HES has not been reported in recent studies. Also, it is found that the use of a combination of COE-LPSP-HDI to establish the optimal size of HRES is not often seen in the current literature. Therefore, this study specifically focuses on several objectives to perform 3E evolution and a comparative study of the proposed HES system using MATLAB simulation. Here the sizing and 3E analysis of the HRES system is presented as an objective function defined as the minimization of COE, NPC, ASC, CO_2_ emission, and LPSP thereby improving PVF, WTF, RF, and HDI. Also case study of the HRES system consists of PV-WTG-BESS-DG configuration. The contributions of the work are summarized as follows:

- A modified version of HHO known as the Modified HHO (mHHO) algorithm is presented in this paper and this is the first such attempt in the HRES design utilizing mHHO for rural electrification.
- The proposed mHHO algorithm with 9 different operators is evaluated on the CEC 2019 benchmark dataset. A statistical test, specifically the Wilcoxon's rank-sum (p-rank) and Friedman tests (f-rank), are employed to compare the modified algorithms against each other and to choose the most efficient among them.
- Developing a Hybrid Renewable Energy System (HRES) by combining PV-WT-BES- DG which provides a very cost-effective and efficient solution for supplying power to a rural region.
- To determine the most cost-effective and feasible HRES configuration, the sizing of HRES components with a comprehensive 3E analysis (Energy, Economic [COE, NPC, and Annual cost] and Ecological [CO_2_ emission]) is conducted by considering technical (LPSP, RF, PV fraction, and Wind fraction) and social (HDI) factors.
- The performance and effectiveness of the proposed mHHO for the HRES system are verified by comparing it to other MH algorithms such as LCA, NBA, MPO, AOA, EDO, and SCA.

**Fig. 2:**
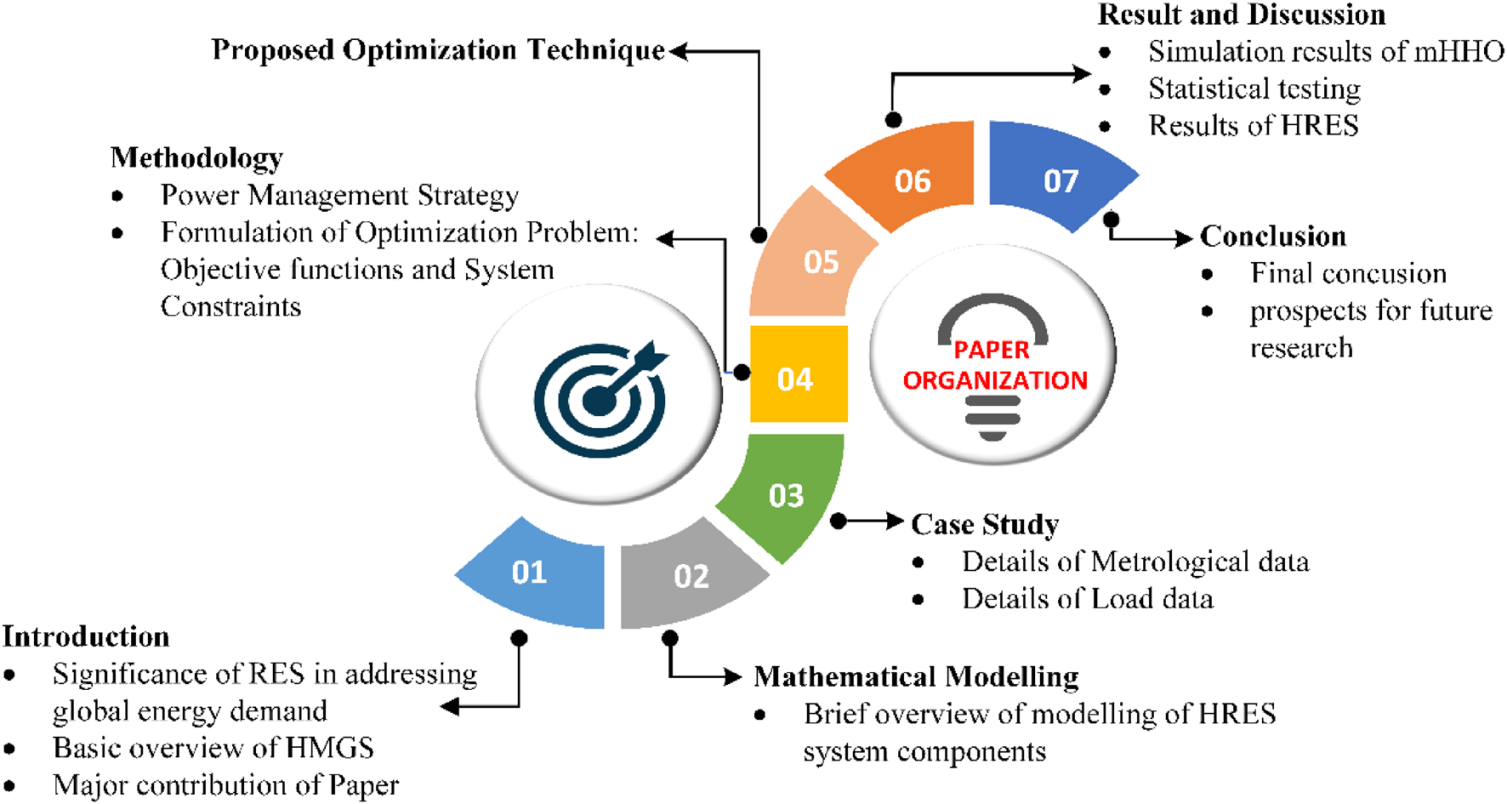
Graphical organization of the paper.

The subsequent sections of the document are organized in the following manner: “Mathematical modeling” of the paper outlines a mathematical model of the various components of an HRES. “Case study” emphasizes the details of the selected location for a case study. “Methodology” describes the methodology which includes a power management strategy and the formulation of an optimization problem, which further consists of two components: objective functions and system constraints. “Proposed optimization technique” demonstrates the basic HHO algorithm and proposed optimization technique mHHO algorithm. “RESULT AND DISCUSSION” is divided into two subsections where the first section analyzes and discusses the simulation outcomes of the mHHO algorithm and the second section addresses the simulation results of HRES using mHHO. Finally, “Conclusion” offers few conclusive findings or remarks. Figure 2 illustrates the detailed structure of the proposed work.

## Mathematical modeling

Hybrid Power Systems (HPS) integrate a minimum of one RES (such as wind, solar, or hydropower) with one or more traditional sources of energy, such as DGs. Due to its reliance on atmospheric conditions as already pointed out, the production of electricity from an RES is unpredictable. Therefore, combining this with conventional power sources and/or battery storage would provide reliable and constant electrical power and in this work such a system is considered as HRES. A HRES is typically made up of generation, distribution, and demand subsystems, which may all change significantly based on a number of factors. Some of the factors are the accessibility of renewable resources, the intended services to be offered, and the demand subsystem. The aforementioned parameters exert a significant influence on the decision-making process, and consequently on both the cost as well as dependability of the system^66^.

**Fig. 3:**
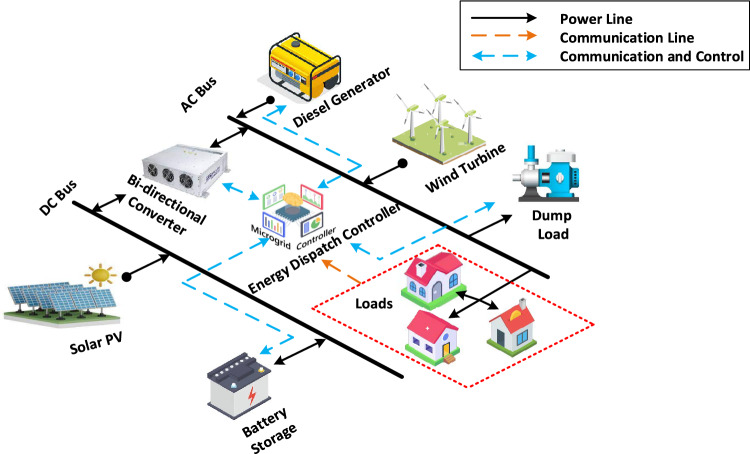
Proposed layout of hybrid energy system.

The design of HRES includes several elements, including a solar PV power generation system, WT power generation system, Power Converters (PC), Battery Storage System (BSS), and DG system which are depicted in Fig. 3. Distributed generation is typically utilized as a final option and is exclusively started when other energy sources are incapable of satisfying the load demand at a specific moment. RESs are expected to offer a consistent supply of electrical power throughout some interval which depends on various factors and optimization of those factors is of utmost interest in this work. The management of distributed energy sources and loads within the HRES network is facilitated through the operation of Local Controllers (LC) which are typically implemented as power electronic converters. Since the operation is controlled by electronic devices, the overall operation of the system is controlled by the Energy Dispatch Controller (EDC) or Micro-grid Controller (MGC) which sends command signals to every of the Local Controllers (LCs). The following subsections describe the mathematical modeling of each component of the proposed system to scrutinize its performance.

### *SolarPV*

The apparoch proposed in this work takes into account two significant parameters, namely solar irradiance as well as ambient temperature, which could potentially affect the power output of solar PV at a certain period of time. The amount of electricity produced by the panels is calculated as a function of solar radiation^67^ and the corresponding equation is given as follows^68^:

1 $$P_{pv} = P_{pv\_r} \times \frac{{G_{T} }}{{G_{T\_STC} }}[1 + \psi_{T} (T_{amb} + (0.0256 \times G_{T} )) - T_{STC} ]$$

where P_pv_ is the output power (Watt) of the solar PV, P_pv_r_ refers to the rated power (Watt) of a PV system under Standard Test Conditions (STC), G_T_ denotes the hourly sun irradiance (W/m^2^) that a solar PV panel surface receives. G_T_STC_ refers to the solar irradiance at STC, which is equivalent to 1000 W/m^2^, $\psi_{T}$ denotes the coefficient of the temperature of solar PV (− 3.7 × 10^−3^ °C^−1^), T_*amb*_ symbolizes the ambient temperature (°C), T_STC_ signifies the temperature of the PV cell at STC, which is typically 25 °C.

### Wind

Wind energy is a renewable and readily available resource that can be utilized to generate electricity. Electricity is generated by Wind Turbines (WTs) through the utilization of the kinetic energy of the wind^69^. The speed of the wind is the primary factor that governs the energy output of a WT. In order to obtain wind speed measurements at a specific hub height, it is necessary to convert the recorded wind speed at a different altitude. This is due to the fact that wind speed is not constant across different altitudes. The conversion process involves utilizing the power law equation of the wind profile, which is expressed in the following Eq. (2)^70^.

2 $$V_{h}(t) = V_{ref}(t) \left( {\frac{{H_{h} }}{{H_{ref} }}} \right)^{\alpha }$$

The equation presented involves the variables V_h_(t) and V_ref_(t), which respectively represent the wind speed (measured in m/s) at the hub height H_h_ (measured in m) of a wind turbine and the reference wind speed (measured in m/s) at the anemometer height H_ref_ (measured in m). The symbol α indicates the friction coefficient, which is also known as the Hellmann exponent wind gradient, or power-law exponent. The variable $\alpha$ is subject to various parameters like as wind speed, temperature, the curvature of the terrain, and altitude above the earth's surface. The standard value of $\alpha$ is 1/7 for the areas with moderate roughness and good exposure^71^. The value of $\alpha$ can be calculated using the following Eq. (3)^72^.

3 $$\alpha = \frac{{0.37 - 0.088\ln \left( {V_{ref} } \right)}}{{1 - 0.088\ln \left( {\frac{{H_{ref} }}{10}} \right)}}$$

The output power P_WTG_(kW/m^2^) from Wind Turbine Generators (WTGs) for a given wind speed V_h_ (m/s) can be determined by using Eq. (4)^73^.

4 $$P_{WTG} = \left\{ \begin{aligned} & 0 & V_{h} < V_{CI} \,or\,V_{h} \ge \,V_{CO} \hfill \\ & P_{WTG\_R} \left( {\frac{{V_{h}^{3} }}{{V_{R}^{3} - V_{CI}^{3} }}} \right) - P_{WTG\_R} \left( {\frac{{V_{CI}^{3} }}{{V_{R}^{3} - V_{CI}^{3} }}} \right) & V_{CI} \le V_{h} <\,V_{R} \hfill \\ & P_{WTG\_R} & V_{R} \, \le \,V_{h} \, \le \,V_{CO} \hfill \\ \end{aligned} \right.$$

where P_WTG_R_ is the rated power of WTG, and V_h_ denotes the wind speed measured at the hub height of a WT. V_R_(m/s), V_CI_, and V_CO_ symbolizes the rated wind speed, cut-in wind speed, and cut-out wind speed of the WT respectively.

### Diesel generator

DGs have been identified as a dependable and cost-effective solution for mitigating energy storage requirements, particularly in rural industries as well as remote regions. DGs consist of compression ignition engines and an alternator that employs non-renewable resources specifically oil to generate electrical power. The utilization of the DG may vary depending on the system, as it can serve as either the primary or supplementary source. DG also serves as a backup source of energy in case of battery depletion during a period of high demand^74^. The quantity of fuel necessary for electricity production is dependent upon variables such as the generator's fuel heat rate efficiency as well as the fuel's heat content. The efficiency of the generator is a function of the load present during operation.

When developing a hybrid system, it is important to take into account the diesel generator's efficiency and fuel consumption per hour. The assessment of fuel consumption in a DG is based on the size of the DG (generator's power output) and the magnitude of the load it encounters. The majority of DGs function lies within the range of 80% to 100% of their designated power output. The fuel consumption (F_DG_ (litre/kWh)) of a DG is computed by utilizing Eq. (5)^75^.

5 $$F_{DG} = \alpha_{1} \times P_{gen} (t)\, + \,\alpha_{2} {\kern 1pt} \times P_{R}$$

where $P_{gen}$ denotes operational or generated power output at time t and P_R_ represents DG power rating of DG in kW respectively. The gradient and intercept coefficient of the fuel curve are both expressed in terms of litre/kWh and denoted by $\alpha_{1}$ and $\,\alpha_{2} {\kern 1pt}$. The values of $\alpha_{1}$ and $\,\alpha_{2} {\kern 1pt}$ have been determined to be 0.246 and 0.0842 respectively for the DG that is used in the design^76^.

### Invertor

DC/AC and AC/DC power converters are essential for systems that utilize both alternating current (AC) and direct current (DC) elements. The solar PV panels and BES, deliver a DC output, however the load being considered demands an AC. The choice of the converter size is determined by the greatest demand for load (P_PLD_). The equation for determining the inverter rating (P_inv_) is as follows^77^:

6 $$P_{inv} (t) = \frac{{P_{PLD} (t)}}{{\eta_{inv} }}$$

where η_inv_ represents the efficiency of the inverter.

### Battery energy storage system

Batteries serve the purpose of storing electrical energy for utilization during instances of power surges and the unavailability of alternative resources. Battery Energy Storage System (BESS) is commonly employed as a means of backup for hybrid stand-alone energy systems with the aim of enhancing their availability and facilitating load leveling for temporary fluctuations. Lead-acid batteries are frequently utilized as a viable alternative for HRESs due to their cost-effectiveness, low Depth of Discharge (DOD), elevated safety standards, recyclability, and rechargeable nature^78^. The capacity of the battery bank is dependent upon two factors such as the need for the electrical load and the projected duration of time at which the BESS is anticipated to provide power to the load. The BESS is expected to provide power to the load only when the RES output is inadequate to satisfy the energy demand. This duration of time is commonly referred to as the Days of Autonomy (DOA). The system's battery capacity C_Bat_ in kW is determined based on the required demand and desired number of DOA, as specified by the following Eq. (7)^79^.

7 $$C_{Bat} \, = \,\frac{{P_{L} \, \times D_{a} \,}}{{DOD\, \times \,\eta_{Bat} \, \times \eta_{inv} }}$$

where $P_{L}$ denotes the load demand (in kilowatts) that needs to be fulfilled by the BESS. $D_{a}$ represents the number of autonomy days, which is typically within the range of 3–5 days. DOD signifies the depth of discharge, which is set at 80%. Furthermore, $\eta_{inv}$ and $\eta_{Bat} \,$ corresponds to the inverter and battery efficiencies, which are 95% and 85%, respectively.

The process of charging as well as draining of a Battery Storage System (BSS) which primarily uses lead acid batteries occurs in a sequential manner. Consequently, the present state of charge of P_BES_ (t) is reliant upon the prior state of charge of P_BES_ (t−1). During the charging period, the updated state of BSS energy may be determined using Eq. (8)^80^.

8 $$P_{BES} (t - 1) = P_{BES} (t)(1 - \delta ) + \left[ {P_{PV} (t) + P_{WT} (t) - \frac{{P_{load} (t)}}{{\eta_{inv} }}} \right]*\eta_{BES}$$

where the symbol δ represents the self-discharge rate, η_inv_ represents the inverter efficiency, and η_BES_ represents the charge efficiency of the battery. When the output power of a RES is insufficient to meet the load demand, the BSS switches into a discharge mode. The updated status of BSS energy for the discharge phase can be determined by (9)^81^.

9 $$P_{BES} (t + 1) = P_{BES} (1 - \delta ) - \left[ {\frac{{P_{load} (t)}}{{\eta_{inv} }} - \left( {P_{PV} (t) + P_{WTG} (t)} \right)} \right] \times \eta_{BES}$$

## Case study

**Fig. 4:**
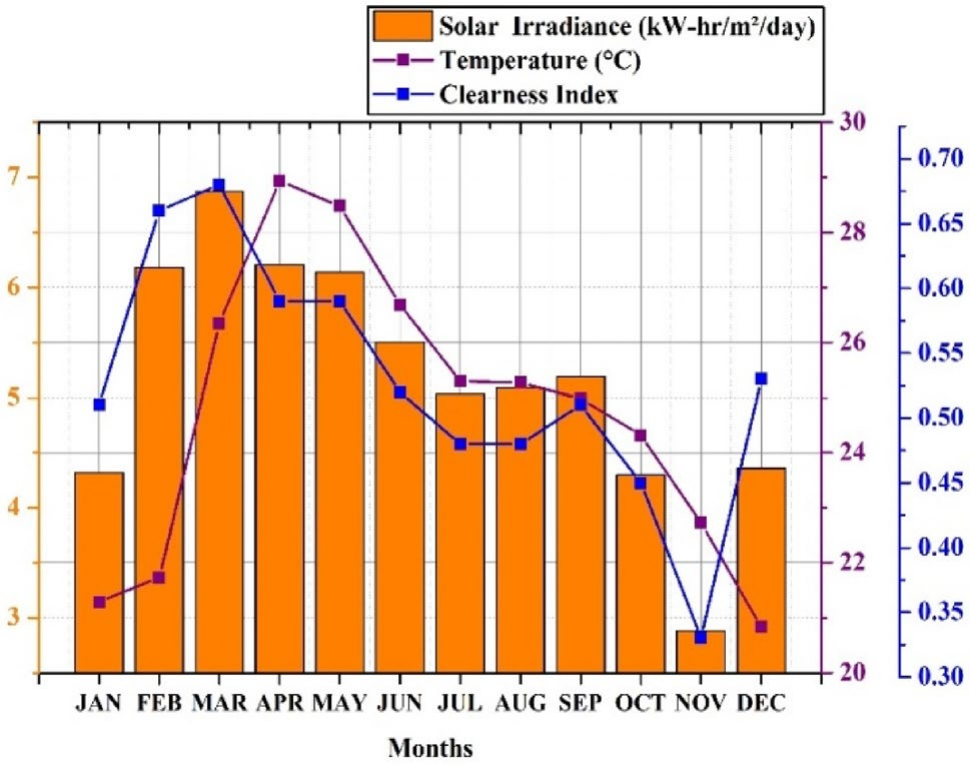
Solar energy data.

**Fig. 5:**
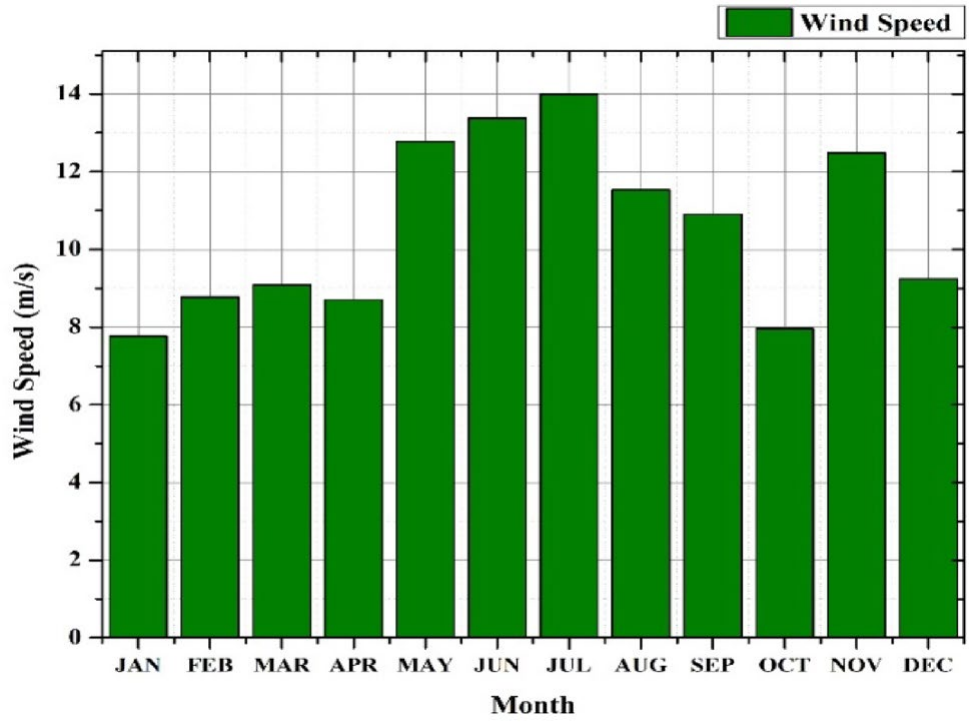
Wind Energy data.

The approach implemented in this study can be used to create a compact independent PV-wind-battery-DG hybrid system which is shown in Fig. 3. This compact design is assumed to fulfill the energy needs of a small community located in a village and can serve as a potential case study location. The selected case study location for this research is Alleri village, situated in close proximity to Vellore city in the Tamil Nadu region of India. The geographical coordinates of the village are around 12.8095 latitude and 78.9913 longitudes. The solar irradiation, temperature, and wind speed data for the site location were collected from the database of NASA Power Data Access viewer website database for the year 2021. The average value of solar irradiation and temperature during the said period are 5.17 (kW-h/m^2^/day) and 24.76 °C respectively. Also, the wind speed range is 5.06 m/s. Figure 4 depicts the hourly solar radiation, temperature, and Clearness Index data of the selected location and this is one of the data sets which is used to model the proposed HRES in this work. The main reason behind selecting the data set is that it has large variance over the period and it is useful to achieve realistic HRES design. This data is obtained from the NASA Power Data Access Viewer website^105^. The hourly wind speed statistic for the selected case study location is shown in Fig. 5.

### Load profile

The load experienced by individuals is influenced by a variety of factors, including geographical conditions, economic circumstances, standard of living, as well as climatic conditions. In the context of usual household consumers, it is common for various electrical appliances to be utilized. These appliances include water pumps, electric irons, ceiling fans, LED lights, pedestal fans, televisions, refrigerators, etc. When assessing the energy consumption of these appliances, it is important to consider the average daily running hours for each appliance, as this provides a more accurate representation of their usage patterns within a household. The analysis of the load profile reveals that the load exhibits continuous variations over the course of the day. To enhance the authenticity or realistic nature of community load, a randomization factor of 10% can be added to the maximum value. The calculation of the total hourly as well as yearly load in kilowatt-hours (kWh) is dependent on the number of residential or domestic devices utilized along with their respective ratings. This calculation is performed using Eq. (10)^82^.

10 $$E_{demand} = \frac{{\left( {\sum\nolimits_{i = 1}^{{Load{\mkern 1mu} type}} {n_{user} {\mkern 1mu} \times P_{rating} \times T} } \right)}}{1000}$$

where $E_{demand}$ denotes the energy or electricity demand (kWh), $n_{user}$ represents the number of loads or equipment and T refers to the time of use. The examination of the load profile of a given area is of greatest significance when it comes to the development of a dependable and effective system customized to that specific region. The determination of battery size and modeling is dependent upon the load profile. Additionally, it is important to note that the reliability of the system is influenced by peak times and consumer behavior. These factors also play a significant role in determining the appropriate sizing of system components and the COE.

**Fig. 6:**
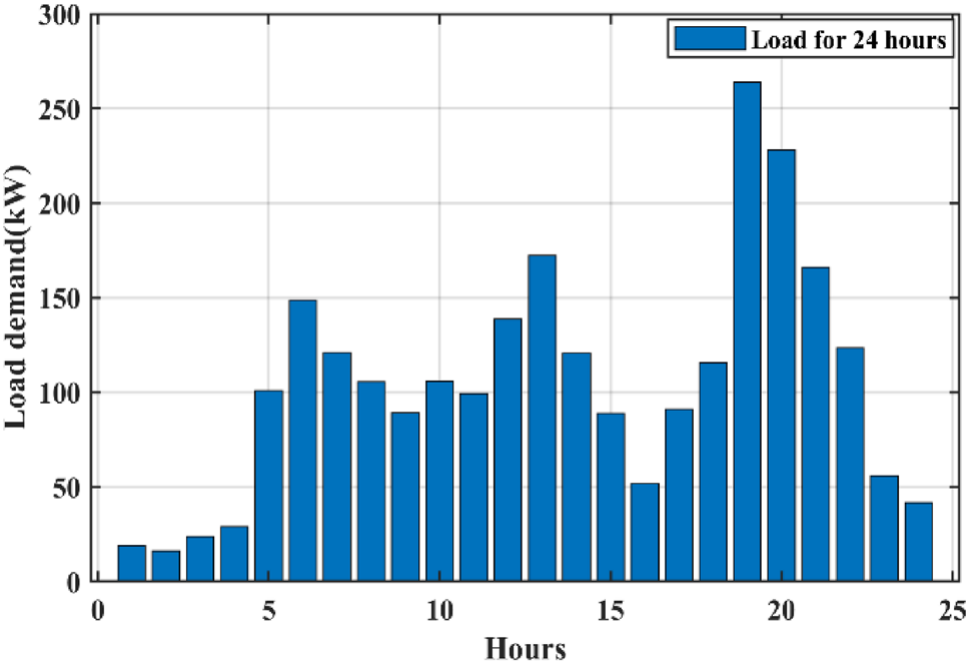
Daily load profile of the entire village.

**Fig. 7:**
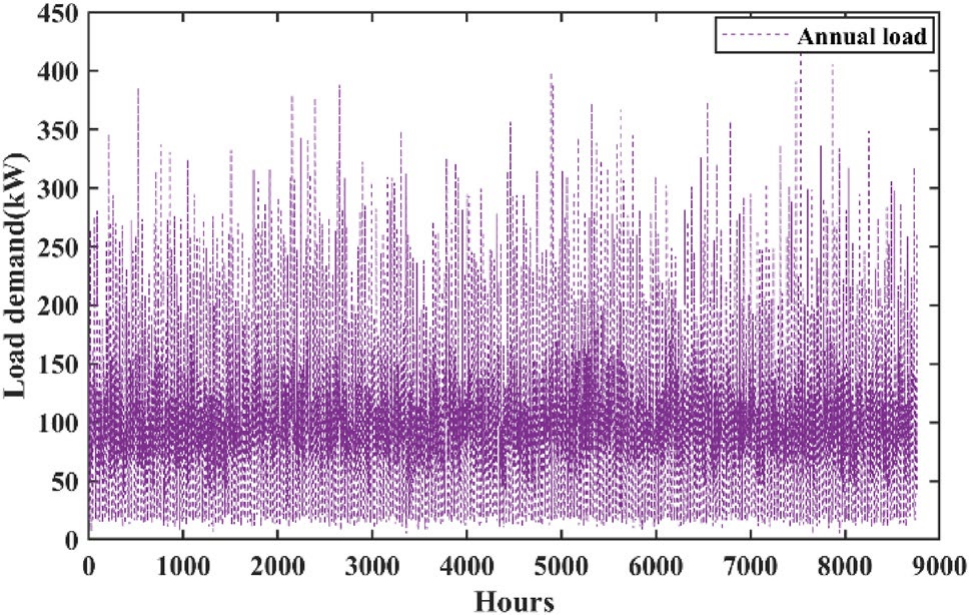
Yearly load profile of the entire village.

The appropriate sizing of the HRES is mostly dependent upon the load requirements, and it signifies the energy consumption over one year. The data related to the electrical consumption of households of the selected case study on an hourly basis over a period of a year is obtained from the HOMER software. Figure 6 shows the daily load profile of the entire village. Figure 7 presents the annual hourly load curve. The daily rate of change in load is taken as 10%, whereas the hourly rate of change in load is considered as 20%.

## Methodology

### Power management strategies

The integration of the Power Management Strategy (PMS) is a crucial aspect of hybrid energy systems. Its primary function is to ensure that a balance is maintained between the power generated by RES and the power required to meet the load demand. This is particularly important in situations where weather conditions are variable and there is a high degree of uncertainty associated with RES. As a result of the restricted amount of power that may be generated by renewable sources, the capacity of the DGs cannot rapidly be raised to satisfy the growing demand. In cases at which the power generated surpasses the demand, it becomes necessary to utilize a dump load which is also known as a diversion load or dummy load to dissipate the surplus energy and protect the storage devices like battery banks from overcharging. The term "dump loads" is most commonly used to refer to deferrable loads, such as electric water heaters and hot air heaters used in residential environments, water pumps, and power resistances. Thus, it is essential to incorporate PMS in the design of such systems. In order to apply an EMS, it is necessary to conduct simulations that take into account the following conditions or approaches.

State I: Battery charging approach: In case, at which overall energy output from RES surpasses the load demand within a specific time frame and the State of Charge (SOC) of the battery has not yet reached its maximum predefined capacity, the battery energy storage system is utilized to consume the excess energy for battery charging. Alternatively, if the aforementioned conditions are not met, approach 2 is initiated.

State II: Energy dumping approach: Analogous to the first approach, in cases at which the overall energy output of RESs exceeds the instantaneous load requirement and the SOC of the battery bank is at its maximum. Thus, in this scenario, the excess power is utilized in dump loads. The implementation of this particular strategy serves to mitigate the risk of battery bank damage resulting from overcharging.

State III: Battery discharging approach: RESs are unable to meet the load demand due to insufficient energy production. In this scenario, the foremost concern is to utilize the energy stored in the battery banks, instead of relying on the functioning of the DG. In this instance, the deficit in the production of electrical energy is compensated for using a battery.

**Fig. 8:**
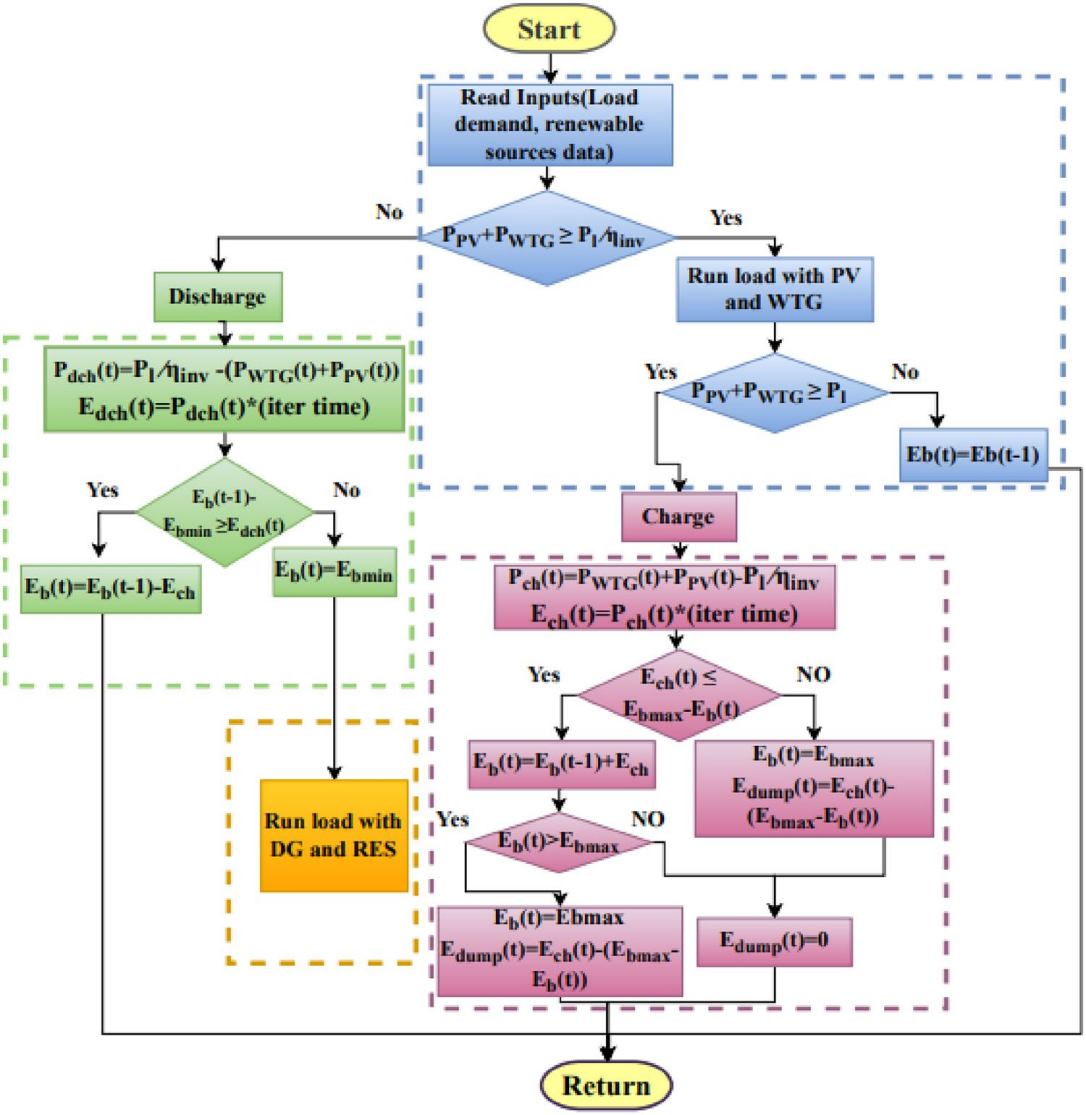
Flowchart of power management system of HRES.

State IV: DG supply approach: In the case that the energy produced by the combined RESs and the energy that has been accumulated in the battery fails to satisfy the energy requirements, the DG is activated in order to compensate for the energy deficit or to provide power to the load and to facilitate the recharging of the battery banks. The proposed dispatch strategies are represented in a simplified flowchart, as shown in Fig. 8.

### Formulation of optimization problems

In order to develop a cost-effective and optimal performance of HRES, it is essential to prioritize the sizing of the various system components. The primary determinants are the ideal sizing of PV systems, WTGs, BESS, and DGs. The utilization of various generation sources along with the incorporation of superior quality components significantly impacts the lifespan of the system, leading to reduced electricity costs for end-users residing in remote regions. The subsequent sub-sections provide details pertaining to the objective function and constraints of the system.

#### Objective function

The lifespan of a system can be significantly impacted by the integration of RESs and the utilization of components of superior quality. This in turn can lead to a reduction in the COE for end-users residing in remote regions.

##### Economic or cost assessment

Conducting an economic analysis is a significant consideration for any executing agency before the establishment of a renewable energy project. Prior to initiating a project, it is essential to conduct a comprehensive evaluation of its viability across various aspects including its economic feasibility. The economic feasibility of an HRES can be assessed through the evaluation of three parameters namely ASC, NPC, and COE.

The Annualized System Cost (ASC) concept is used for economic evaluation. The solution with the lowest ASC is regarded as the most optimal provided it meets all other requirements and characteristics. The ASC can be described by the following Eq. (11)^70^.

11 $$ASC = f\left( {N_{PV} C_{PV} + N_{WT} C_{WT} + N_{BES} C_{BES} + P_{INV} C_{INV} + N_{DG} C_{DG} } \right)$$

C_PV_ is the cost of a solar PV panel per kilowatt, C_WT_ is the cost of a wind turbine per kilowatt, C_BES_ is the cost of a battery per unit, and C_INV_ is the cost of an inverter per kilowatt. C_INV_ represents the cost of the inverter, whereas P_INV_ indicates the rating of the inverter. Each component's cost includes capital, replacement, operation and maintenance, fuel, and salvage expenses.

Furthermore, this research aims to decrease the total Net Present Cost (NPC) of the proposed HES in order to minimize the COE while maintaining optimal energy flow. The total NPC is a component that plays a significant role in the development of energy, and COE is the metric that is used for examining hybrid systems. The Total Net Present Cost (NPC) is a financial measure that can be employed to evaluate the economic feasibility of an investment project. The total net present cost (TNPC) can be determined based on the assessment of HRES^71^. The cumulative discounted cash flows throughout the year of the project's lifecycle are added together to determine the total NPC^79^. The total NPC may be determined using Eq. (12)^77^.

12 $$NPC = \frac{ASC}{{CRF}}$$

As pointed out earlier, the primary aim of this optimization problem is to minimize the COE by identifying the most cost-effective optimal size of the HRES component that can efficiently fulfill the energy demands. The present study incorporates the cost associated with the operation of HES, which involves the costs of PV array, WT, inverter, and DG. It is referred to as the cost per unit of electric power or the constant price per unit of energy. It is determined by employing the following Eq. (13)^83^.

13 $$COE = \frac{{NPC \times {\mkern 1mu} CRF}}{{\sum\nolimits_{h = 1}^{8760} {P_{load} } }}$$

where the variable P_load_ is utilized to indicate the hourly consumption of electricity or the aggregate load that has been served. The calculation of the NPC involves determining the present value of the total capital investment as well as operational costs incurred over the entire lifespan of the project. The parameter CRF is an abbreviation that stands for Capital Recovery Factor, which is a ratio employed to measure various components in relation to the current interest rate during a specific period which is given in Eq. (14)^33^.

14 $$CRF(\gamma ,\tau ) = \frac{{\gamma (1 + \gamma )^{\tau } }}{{(1 + \gamma )^{\tau } - 1}}$$

15 $$\gamma = \frac{\gamma ^{\prime} - f}{{1 + f}}$$

where the variables $\gamma$ and τ represent the actual or real discount rate and the number of years. $\gamma ^{\prime}$ represents the nominal interest rate and $f$ denotes the annual inflation rate respectively.

##### Reliability assessment

The assessment of system reliability is of greatest significance as unpredictable weather can significantly affect the performance of power generation equipment. An effective power supply system necessitates minimal or ideally zero loss of load. The Loss of Power Supply Probability (LPSP) is a statistical metric that determines the possibility of power supply failure. This failure can be attributed to either insufficient renewable resources or technical issues that prevent meeting the demand for power. LPSP serves as an indicator of the reliability of the hybrid systems in fulfilling the power demand. It can be defined as follows^84^.

16 $$LPSP\, = \sum\limits_{t = 1}^{{t_{f} }} {\frac{{(P_{load} - P_{PV} - P_{WTG} + P_{BES} + P_{DG} )}}{{P_{Load} \left( t \right)}}}$$

where $t_{f}$ represents the final time or operating period which is typically 8760 h for one year, P_Load_ indicates total or overall electric energy demand and DPS is the deficiency of power supply. Chronological simulation and probabilistic approaches are the two ways to compute LPSP. The first method makes utilization of time-series data over a certain period, while the second relies on the energy accumulative impact of an energy storage device. LPSP has values ranging from 0 to 1. When the power generated equals the load demand, the load is fully supplied by the generated power and the LPSP value is 0. The LPSP value of 1 indicates that the load is fully unsatisfied i.e. load demand is not met by the generated power.

##### System constraints

This study introduces the concept of the Renewable Factor (RF) as a means of evaluating and comparing the energy output derived from renewable sources with respect to the energy produced by DGs. Similarly PV fraction, WT fraction also measures the respective energy output aganist energy produced by DGs. The ideal system is one that only uses renewable resources which is demonstrated by the variable RF and in this case the value is 100%. Although, the RF = 0% demonstrates that the energy produced by a DG is equal to the energy produced by renewable resources. RF, PV fraction (PVF) as well as WT fraction (WTF) are determined by employing Eqs. (17), (18) and (19)^85^.

17 $$RF(\% ) = \left( {1 - \frac{{P_{DG} }}{{P_{PV} + P_{WTG} }}} \right) \times 100$$

18 $$PV\,fraction = RF \times \left[ {\frac{{P_{PV} }}{{P_{PV} + P_{WT} + P_{DG} }}} \right]$$

19 $$WT\,fraction = RF \times \left[ {\frac{{P_{WT} }}{{P_{PV} + P_{WT} + P_{DG} }}} \right]$$

20 $$N_{PV}^{\min } \le N_{PV} \le N_{PV}^{\max }$$

21 $$N_{WTG}^{\min } \le N_{WTG} \le N_{WTG}^{\max }$$

where,$N_{PV}^{\max }$,$N_{PV}^{\min }$, $N_{WTG}^{\min }$, $N_{WTG}^{\max }$ are the number of maximum and minimum of PV and WTG respectively.

##### Social parameter assessment

The Human Development Index (HDI_y,z_) is commonly utilized as a statistical tool for assessing a country's overall growth in its economic as well as social indicators. The HDI is determined by combining the values of the elements "y" along with the control approach "z" using a specific Eq. (22)^86^. The HDI is a numerical metric used to assess the level of continuous advancement in a country or region. It relates to the overall well-being of persons which includes the services, physical infrastructure, and resources available to them. This research incorporates the HDI into the objective function to provide a design solution that considers the well-being of society. Excess or wasted energy may improve the overall quality of life in society. The HDI is calculated in the following manner^87^.

22 $$HDI_{y,z} = 0.0978\,\ln \left[ {\frac{{\left( {P_{load} + \min (F_{\max \_DP} \times P_{Dump} ,F_{\max \_load} \times P_{load} )} \right)}}{{\varepsilon_{pop} }}} \right] - 0.0319$$

where F_max_DP_ represents the percentage of the greatest energy that is dissipated. F_max_load_ is the limiting factor for the extra load that the available power can handle, while ℇ_pop_ indicates the number of persons benefiting from the hybrid system.

## Proposed optimization technique

**Fig. 9:**
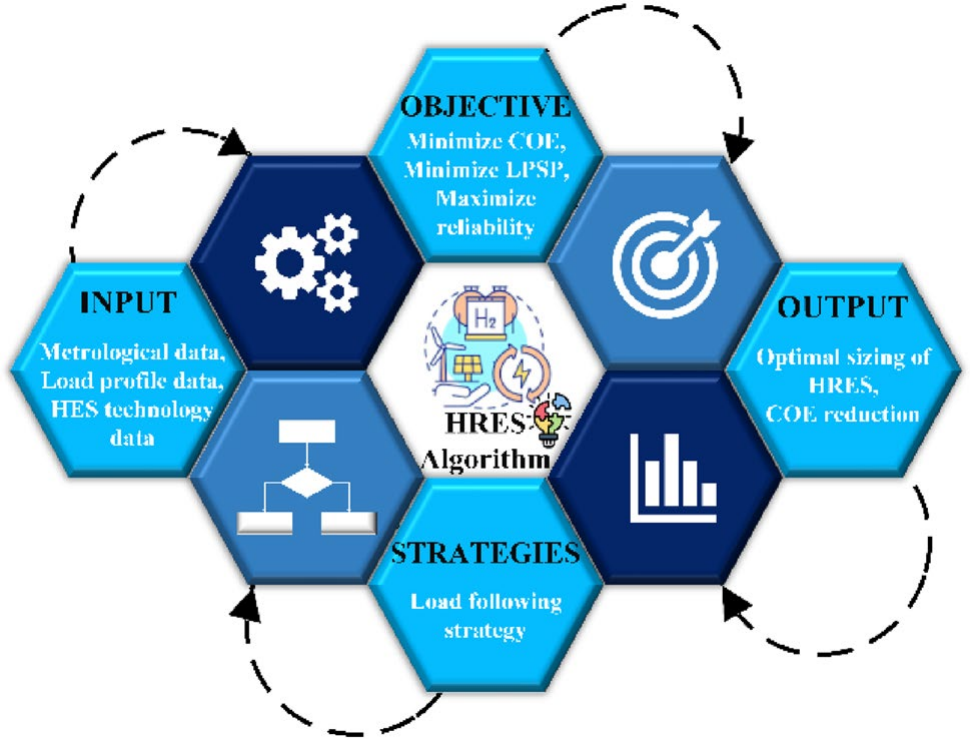
Process or Flow diagram of HRES optimization problem.

The optimization of hybrid systems through the implementation of an EMS can be classified into four main categories. These categories include load supply optimization, which is a simple optimization method dependent on load as well as power. Technical optimization is another category that aims to improve the operation and durability of the hybrid system. Economic optimization on the other hand focuses on reducing maintenance costs through an optimization algorithm based on cost reduction function. Lastly, techno-economic optimization is a more complex category that employs an optimization algorithm depending on the objective function. As already said, this research employs a 3E analysis to evaluate the energy, ecological, and economic aspects with social, and technical factors assessment of HRES. To address these issues, intelligent methods like MH algorithms are the best way to find a solution. Figure 9 illustrates the problem-solving methodology adopted in this work using the mHHO optimization technique. The flow chart (Fig. 9) depicts the usage of real-time input datasets to evaluate objective functions, namely technical, economic, environmental, and social factors. The primary purpose is to achieve output segments that include the least COE, NPC, and ASC, minimum LPSP, and maximum reliability of the system while maximizing the HDI.

### Harris Hawk optimization algorithm (HHO)

The HHO algorithm takes inspiration from the hunting as well as capturing strategies used by hawks. The objective of this method is to initiate an assault on a target, often a rabbit, by the coordinated efforts of many hawks approaching from various angles, with the intention of catching the prey unaware and unprepared. The typical HHO method may be subdivided into two fundamental stages, namely exploration and exploitation. The exploitation stage consists of two distinct stages, namely the hard passage and the soft passage^50^.

#### Exploration

Exploration is often regarded as a global search mechanism within the context of optimization algorithms. During this stage, the algorithm systematically investigates the search space in order to identify and evaluate potential solutions that exhibit desirable qualities. In the context of HHO, the Harris hawks exhibit a behavior of perching in different places without a specific pattern, using two distinct approaches to identify potential prey or rabbit. Consider that q is a probability for both the exploration in randomly placed tall trees as well as the exploration based on the location of the rabbit and other hawks as indicated in the following Eq. (23)^88^.

23 $$X(t + 1) = \left\{ \begin{gathered} X_{rand} (t) - r_{1} \left| {X_{rand} (t) - 2r_{2} X(t)} \right|\quad q \ge 0.5 \hfill \\ (X_{prey} (t) - X_{m} (t)) - r_{3} (LB + r_{4} (UB - LB))\quad q < 0.5 \hfill \\ \end{gathered} \right.$$

where t represents the number of iterations.$X(t)$ denotes the current location of the Hawk, while $X(t + 1)$ representing the position of the Hawk in the subsequent iteration. Additionally,$X_{prey}$ signifies the current position of the rabbit. The variable $X_{rand} (t)$ represents a stochastic location for the hawk. The variable $X_{m} (t)$ represents the average location of the hawk's current location, which is determined using Eq. (24). The lower and upper limits of variables are denoted as LB and UB. The factors $r_{1}$,$r_{2}$,$r_{3}$,$r_{4}$ and q are assigned random values. The haw's position is given by the following equation

24 $$X_{m} (t) = \frac{1}{N}\sum\limits_{i = 1}^{N} {X_{i} (t)}$$

where $X_{i} (t)$ specifies each hawk's position in iteration t, and N stands for the overall number of hawks. The transition from exploration to exploitation is dependent upon the escaping energy of prey. In order to represent the process of transitioning from exploration to exploitation, the energy level of prey is conceptualized and represented in a mathematical model as follows.

25 $$E = E_{0} *E_{1}$$

26 $$E = 2E_{0} \left( {1 - \frac{t}{T}} \right)$$

where E represents the prey's escaping energy, E_1_ denotes the linearly decreasing factor with a range between 2 to 0, T denotes the maximum number of iterations, and E_0_ signifies the beginning state of the prey's energy. Exploration occurs when $\left| E \right| \ge 1$ but exploitation takes place in subsequent stages when $\left| E \right| < 1$.

#### Exploitation

Exploitation is often seen as a localized search in comparison to the exploration stage. The algorithm attempts to minimize big jumps inside the search space while simultaneously refining the outcome during this particular phase. There are four internal phases to the exploitation phase, which are based on the level of escaping energy (E) and the probability (r) associated with the successful escape of the prey (r).

- Soft besiege

During this particular stage, when $\left| E \right| \ge 0.5$ and $r \ge 0.5$ the prey has sufficient energy and attempts to escape by unpredictable and deceptive movements. However, ultimately it is unable to successfully escape. During these efforts, the Harris' hawks perform a strategic movement of circling the rabbit softly in a gradual manner, aiming at causing exhaustion before executing an unexpected attack. This behavior is represented by using the following Eq. (27).

27 $$X(t + 1) = \Delta X(t) - E\left| {JX_{prey} (t) - X(t)} \right|$$

28 $$\Delta X(t) = X_{prey} (t) - X(t)$$

29 $$J = 2(1 - r_{5} )$$

where $\Delta X(t)$ represents the difference among the position vector of the prey and its current position at iteration t. Additionally, $r_{5}$ denotes a randomly generated value that falls within the interval (0,1). The variable J denotes the random jumps made by the prey all over the process of escape.

- Hard besiege

During this particular stage, the prey's energy is reduced to a point where it is unable to escape, denoted by $\left| E \right| < 0.5,\,\,r > 0.5$. Consequently, the hawk employs a strategy including hard circles in order to capture the prey, as shown in Eq. (30).

30 $$x(t + 1) = x_{prey} (t) - E\left| {\Delta x(t)} \right|$$

- Soft besiege with progressive rapid dives

The prey has sufficient energy to effectively escape when $\left| E \right| \ge 0.5$ but $r < 0.5$, and a mild besiege is still built before the surprise attack. In comparison to the prior situation, this technique is more sensible. Observing hawk behaviors, it is hypothesized that they may choose the optimal dive to grab prey in competitive scenarios. Thus, to softly besiege, it is assumed that the hawks could assess (decide) their next step using the following Eq. (31)

31 $$Y = x_{prey} (t) - E\left| {Jx_{prey} (t) - x(t)} \right|$$

However, as a result of the deceptive and unpredictable movement pattern shown by the prey, the Hawk adjusts its present location to position (Z) by following Eq. (32).

32 $$Z = Y + S \times LF(D)$$

where D denotes the dimension of the problem, the random vector is represented as S with dimensions $(1 \times D)$, and the levy flight function is denoted as LF as stated by the following Eq. (33).

33 $$LF(x) = 0.01 \times \frac{v \times \sigma }{{\left| v \right|^{{\frac{1}{\beta }}} }}$$

34 $$\sigma = \left( {\frac{{\gamma (1 + \beta ) \times \sin \left( {\frac{\pi \beta }{2}} \right)}}{{\gamma \left( {\frac{1 + \beta }{2}} \right) \times \beta \times 2^{{\left( {\frac{\beta - 1}{2}} \right)}} }}} \right)^{{\frac{1}{\beta }}}$$

where u and v represent random variables that are uniformly distributed between 0 and 1. The constant β is assigned a default value of 1.5. Therefore, the ultimate approach of updating the locations of hawks during the soft besiege phase may be executed using the following Eq. (35).

35 $$x(t + 1) = \left\{ \begin{gathered} Y\quad \,if\,F(Y) < F(x(t)) \hfill \\ Z\quad if\,F(Z) < F(x(t)) \hfill \\ \end{gathered} \right.$$

- Hard besiege with progressive rapid dives

During this phase, the prey may have a limited chance of escape due to a significant fall in its energy, denoted as $\left| E \right| < 0.5$, and r < 0.5. Consequently, an effective besiege is established prior to executing a sudden attack in order to capture as well as kill the prey. The hawk reduces the circle of its hunting area and adjusts its locations by (35). The locations denoted as (Z) and (Y) are defined by using Eqs. (32) and (36), respectively.

36 $$Y = x_{prey} (t) - E\left| {Jx_{prey} (t) - x_{m} (t)} \right|$$

With respect to the described methodology above, there exist several factors that require optimization in order to enhance performance. In the modified method which is described in the next section, the escaping energy of the prey is optimized with 9 different operators which are called as inertial weights. The detailed description of energy change with respect to nine different operators is described below.

### Modified Harris Hawk optimization algorithm (mHHO)

The present work introduces a new approach, namely the Modified Harris Hawk Optimization Algorithm (mHHO), to address the sizing as well as the techno-economic assessment of HRES. The mHHO algorithm is capable of transitioning from a phase of exploration to an exploitation phase, and thereafter adapting its exploitative behaviors dependent on the energy level of the prey's attempts to escape or the prey's escaping energy. In the context of the HHO algorithm, the concept of energy is often linked to the fitness or objective function which undergoes optimization. The search process may experience a decreasing exploration–exploitation balance as the energy component decreases. According to Gauri Sahoo et al.^89^, the transition from the exploration to the exploitation phase is in the context of the prey's escape energy value inside the HHO algorithm. Excessive exploration of the search region might bring a degree of unpredictability or randomness and could lead to being stuck in local minima. In addition, excessive exploitation may lead to reduced levels of unpredictability and could prevent the achievement of optimal outcomes. Hence, an appropriate balance needs to be conserved between the exploration and exploitation phases during the search process^50^. In order to achieve the balance in the current work, various operators are being primarily used for updating the position. In order to symbolize the decreasing energy factor in the HHO system and to optimize the escaping energy one can make use of these operators to generate mHHO algorithms. In this work, the HHO algorithm is modified using 9 different operators as shown in Table 2. The mathematical model or the formula of the operators is provided to further know its behaviors in optimizing the escaping energy. Incorporating the formulae given for various operators in Table 2 on Eq. (24), the new escaping energy of the prey can be evaluated. Once the expected energy change is obtained using the respective operators, the performance of the algorithm is evaluated effectively.

**Table 2:** Different operators used for improving the HHO algorithm.

S. no.	Operators or factor	Formula	References
1	Linear decreasing factor	$\beta_{k} = \beta_{\max } - \left( {\frac{{\beta_{\max } - \beta_{\min } }}{{T_{\max } }}} \right) \times t$	^90,91^
2	Chaotic	$\beta = (\beta_{\max } - \beta_{\min } ) \times \frac{{T_{\max } - t}}{{T_{\max } }} + \,\beta_{\min } \times Z,\,\,\,Z = 4 \times Z \times (1 - Z)$	^92^
3	Natural exponent	$\beta (iter) = \beta_{\min } + \left( {\beta_{\max } - \beta_{\min } } \right)\exp \left[ { - \frac{t}{{\left( {{{T_{\max } } \mathord{\left/ {\vphantom {{T_{\max } } {10}}} \right. \kern-0pt} {10}}} \right)}}} \right]$	^93^
4	Random	$\beta = 0.5 + \frac{rand()}{2}$	^94^
5	Oscillating	$\beta (iter) = \frac{{\beta_{\max } + \beta_{\min } }}{2} + \frac{{\beta_{\max } - \beta_{\min } }}{{2}}\times \cos \left( \frac{2\pi t}{P} \right),\,\,\,P=\left( \frac{2{{S}_{1}}}{3+2k} \right)$	^95^
6	Simulated annealing	$\beta_{k} = \beta_{\min } + \left( {\beta_{\max } - \beta_{\min } } \right) \times \lambda^{{\left( {k - 1} \right)}} ,\,\,\lambda = 0.95$	^96^
7	Sigmoid decreasing	$\beta_{k} = \frac{{\left( {\beta_{\max } - \beta_{\min } } \right)}}{{\left( {1 + e^{ - u(k - n \times gen)} } \right)}} + \beta_{\min } ,\,\,u = 10^{{\log (\left( {gen} \right) - 2)}}$	^97^
8	Sigmoid increasing	$\beta_{k} = \frac{{\left( {\beta_{\max } - \beta_{\min } } \right)}}{{\left( {1 + e^{u(k - n \times gen)} } \right)}} + \beta_{\min } ,\,\,u = 10^{{\log (\left( {gen} \right) - 2)}}$	^98^
9	Chaotic random	$Z = 4 \times Z \times (1 - Z),\,\,\beta = 0.5 \times rand() + 0.5 \times Z$	^92^

When incorporating these different operators on HHO, the transition from exploration to exploitation phase in the HHO algorithm is achieved. This further helps to achieve optimal solutions. The optimal solution and functioning of the modified HHO (mHHO) using these 9 different operators are tested on 10 different problems proposed by CEC 2019 to demonstrate or show its effectiveness. Moreover, from the implementation of the 9 operators on CEC 2019, one can select the best operators for sizing and techno-economic analysis of HRES.

## Results and discussion

This section presents a detailed examination of the efficacy of the proposed mHHO algorithm and the performance assessment of the HRES system employing mHHO and other MH algorithms. This section is divided into two parts. The first subsection focuses on the proposed Modified Harris Hawks Optimization (mHHO) algorithms. It performs a comparative study among nine different versions of the algorithm using CEC 2019 benchmark functions. This analysis helps identify the optimal mHHO version. Subsequently, this optimal mHHO is compared against various other MH algorithms. The second subsection then evaluates the effectiveness and robustness of the chosen mHHO algorithm. Here, the algorithm is applied to a Hybrid Renewable Energy System (HRES) optimization problem and its performance is compared with other MH algorithms such as Chinese Pangolin Optimizer (CPO)^99^, Arithmetic Optimization Algorithm (AOA)^100^, Exponential distribution optimizer (EDO)^101^, Liver Cancer Algorithm (LCA)^102^, Novel Bat Algorithm (NBA)^103^, and HHO^50^. The parameter settings for every approach are obtained from the standard sources and are shown in Table 3 for ease of analysis and reference. All the simulations are performed by using MATLAB: 2021a on VivoBook_Asus having an Intel core i5 processor (Intel(R) Core(TM) i5-1035G1), 8 GB RAM, and Windows 11 ultimate operating system.

**Table 3:** Details of various algorithm parameters.

Algorithm	Parameters
NBA^103^	$\alpha =\gamma =0.9, {f}_{min}=0, {f}_{max}=1.5, {A}_{0}\in \left[\text{0,2}\right],{r}_{0}\in \left[\text{0,1}\right], G=10, P\in \left[0.5, 0.9\right], w\in \left[0.4, 0.9\right], C\in \left[\text{0.1,0.9}\right],\theta \in [\text{0.5,1}]$
LCA^102^	$\alpha \in \left[\text{0,1}\right],p=\frac{2}{3}, \beta =1.5$
AOA^100^	$\alpha =5, \mu =0.5$
EDO^101^	$\alpha =0.5,\Phi =\left[\text{0,1}\right], f=[-1, 1]$
MPO^99^	$\beta =1.5, {C}_{1}=\text{Linearly decreasing }\left[\text{2,0}\right],\text{ Q}=100$
DE^60^	*F* = 0.5; *CR* = 0.9
SCA^65^	${r}_{1}$= decreases linearly [2,0], ${r}_{2}$ = [0,2 $\pi$], ${r}_{3}$>1 and ${r}_{3}$<1, ${r}_{4}$= [0,1]
HHO^50^	*q* > 0.5; *Eo* = Linearly decreasing [2,0]
mHHO	*q* > 0.5; *Eo* = Natural exponent inertia weight

### mHHO performance analysis

In order to evaluate the effectiveness of the proposed algorithm, the mHHO has been implemented on a total of 9 distinct benchmark test functions^104^. To substantiate the performance, the statistical tests namely Friedman's rank test (f-rank test) and convergence profiles, are used to demonstrate the improved performance of the proposed algorithm. It is important to note that the calculations have been derived from a total of 51 runs, ensuring a robust and reliable assessment of the algorithms' characteristics**.** For fare comparison, maximum iteration and population size of all these algorithms are set to 500 and 50 respectively. The f-rank test includes assigning a distinct rank to each algorithm being compared based on its performance. The results of the f-rank for each benchmark function are presented in Table 4 for each function separately. The overall total f-rank has been determined by considering the performance across all test functions and is also shown in Table 4. Also, all mHHO are compared in terms of mean and standard deviation values.

**Table 4:** Comparison of simulation outcomes of HHO algorithm with different inertia weights (CEC 2019 benchmark functions).

Function	Parameters	HHO-Chaotic	HHO-NatExpo	HHO-Randam	HHO-LinDec	HHO-Oscilla	HHO-SimAnn	HHO-SigDec	HHO-SigInc	HHO-ChaoRand
F1	Mean	5.27E + 04	4.85E + 04	4.96E + 04	5.10E + 04	5.10E + 04	5.01E + 04	5.06E + 04	4.97E + 04	4.99E + 04
	std	1.62E + 04	5.34E + 03	6.14E + 03	6.27E + 03	5.17E + 03	4.08E + 03	1.37E + 04	5.50E + 03	5.50E + 03
	f-rank	9	1	2	7	8	5	6	3	4
F2	Mean	17.355	17.352	17.356	17.358	17.355	17.3586	17.352	17.358	17.353
	std	7.141 E-03	1.55E-03	7.9234 E-03	7.50E-03	8.089 E-03	9.94E-03	6.258 E-03	7.151 E-03	7.15E-03
	f-rank	4	1	6	9	5	7	2	8	3
F3	Mean	12.702	12.702	12.702	12.702	12.702	12.702	12.702	12.702	12.702
	std	4.35E-06	4.61E-06	1.63E-06	4.72E-06	1.49E-06	2.03E-06	5.17E-06	3.48E-06	3.48E-06
	f-rank	6	7	2	8	1	3	9	4	4
F4	Mean	1.88E + 02	1.42E + 02	2.18E + 02	1.57E + 02	2.65E + 02	2.93E + 02	1.88E + 02	2.39E + 02	1.64E + 02
	std	82.171	81.696	89.677	58.728	1.12E + 02	1.24E + 02	67.1858	73.485	73.485
	f-rank	5	1	6	2	8	9	4	7	3
F5	Mean	2.4027431	2.0937908	2.1572281	2.2820113	2.2852693	2.18136263	2.202577	2.4387081	2.3815722
	std	6.26E-01	0.5675478	0.5143128	5.24E-01	4.80E-01	5.21E-01	0.469707	6.13E-01	6.13E-01
	f-rank	8	1	2	5	6	3	4	9	7
F6	Mean	8.651	8.883	9.08	9.087	8.975	8.985	10.025	9.108	8.782
	std	1.02	1.242	1.142	1.158	1.259	1.205	1.1131	1.178	1.178
	f-rank	1	3	6	7	4	5	9	8	2
F7	Mean	4.21E + 02	3.70E + 02	4.04E + 02	3.41E + 02	4.64E + 02	4.51E + 02	4.39E + 02	4.41E + 02	4.58E + 02
	std	2.59E + 02	2.16E + 02	2.22E + 02	1.76E + 02	2.57E + 02	2.04E + 02	2.30E + 02	2.61E + 02	2.61E + 02
	f-rank	4	2	3	1	9	7	5	6	8
F8	Mean	5.74	5.768	5.822	5.769	5.835	5.743	5.868	5.825	5.784
	std	0.428531	0.493972	0.537861	0.45253	0.58388	0.5201116	0.60338	0.517659	0.517659
	f-rank	1	3	6	4	8	2	9	7	5
F9	Mean	3.508	2.849	3.549	2.998	3.785	3.881	2.872	3.673	3.175
	std	0.468687	0.304677	0.502241	0.3791	0.47093	0.6641043	0.25398	0.446261	0.4462617
	f-rank	4	1	6	3	8	9	2	7	4
F10	Mean	20.097315	20.057686	20.074506	20.116689	20.118034	20.1712138	20.298481	20.15501	20.054089
	std	0.0903949	0.0719156	0.0820493	0.1370119	0.0738216	0.09275655	0.1160463	0.0549953	0.0749953
	f-rank	3	1	4	5	7	8	10	6	2
Overall f-rank value		4.5	2.1	4.3	5.1	6.4	5.8	6	6.5	4.2
Overall f-rank		3	1	4	5	8	7	6	9	2

After observing the results in Table 4, the performance of HHO_NatExpo is superior on all benchmark functions, since it achieved the highest overall ranking among all the tested algorithms. According to the results presented in Table 4, HHO-LogDec produces superior performance compared to other cases when applied to functions F1, F4, as well as F9. For function F3, HHO-Oscilla inertia weight shows better results as compared with the other algorithms. HHO-SigDec shows superior results as compared to the other operators' results for the function F5. For functions F6 and F8, the HHO-Chaotic operator performs better as compared to others. In the context of function F7, it has been observed that the utilization of HHO-LinDec produces superior performance in comparison to other approaches. HHO-ChaoRand outperforms competitors for functions F10. The results are also validated for each case using an f-rank statistical test.

**Fig. 10:**
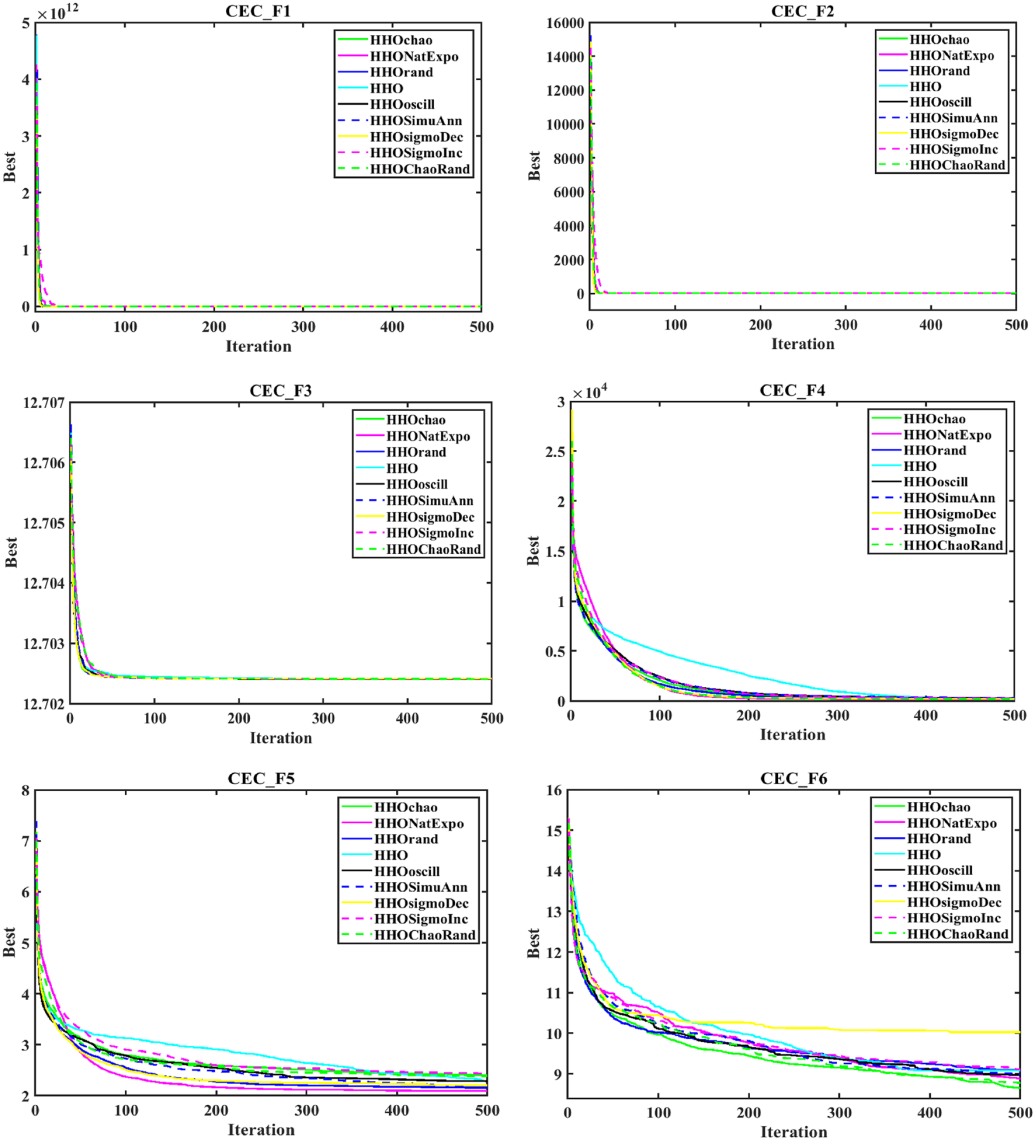

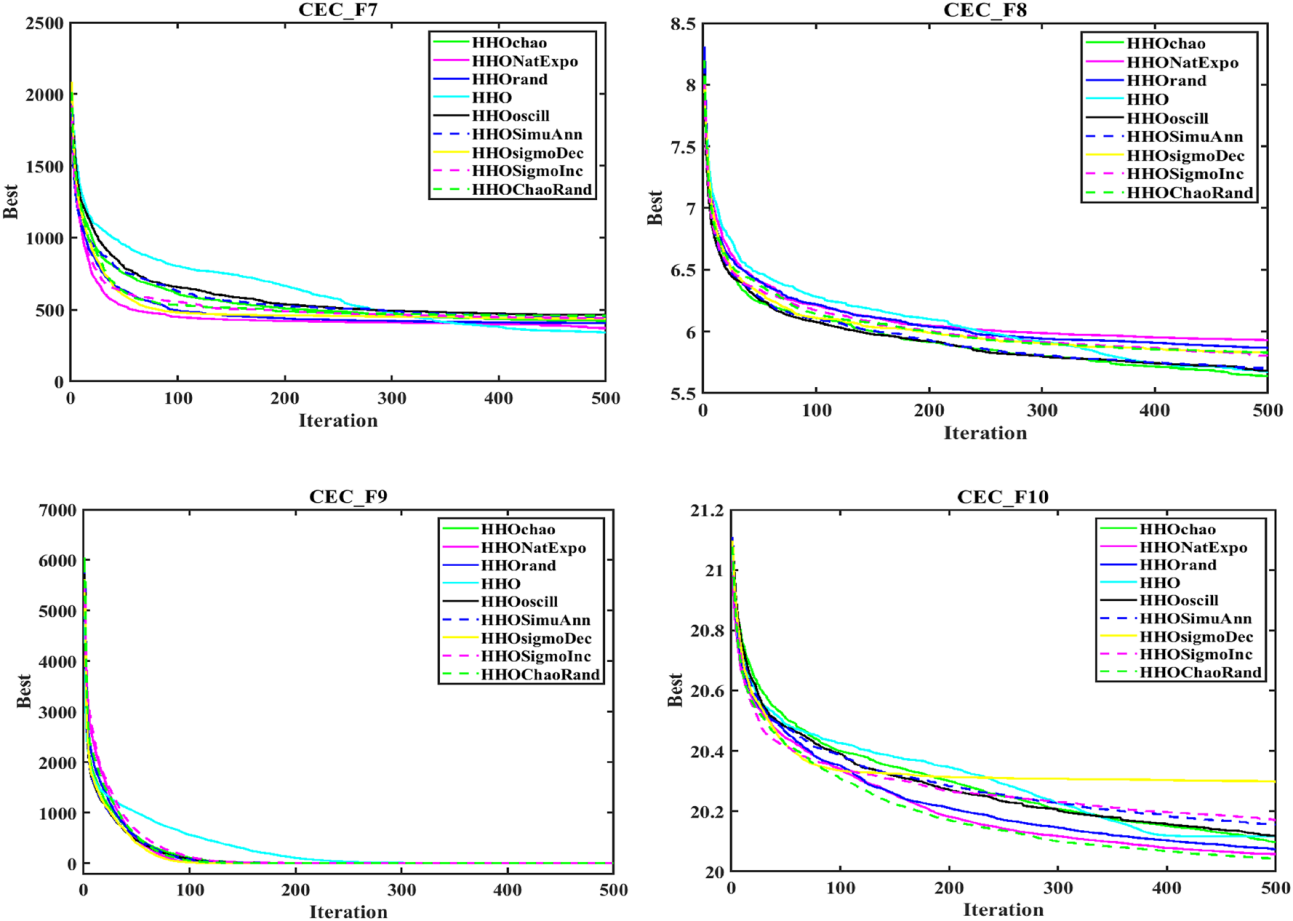
Convergence profile of mHHO with each operator for all benchmark functions (F1–F10).

**Fig. 11:**
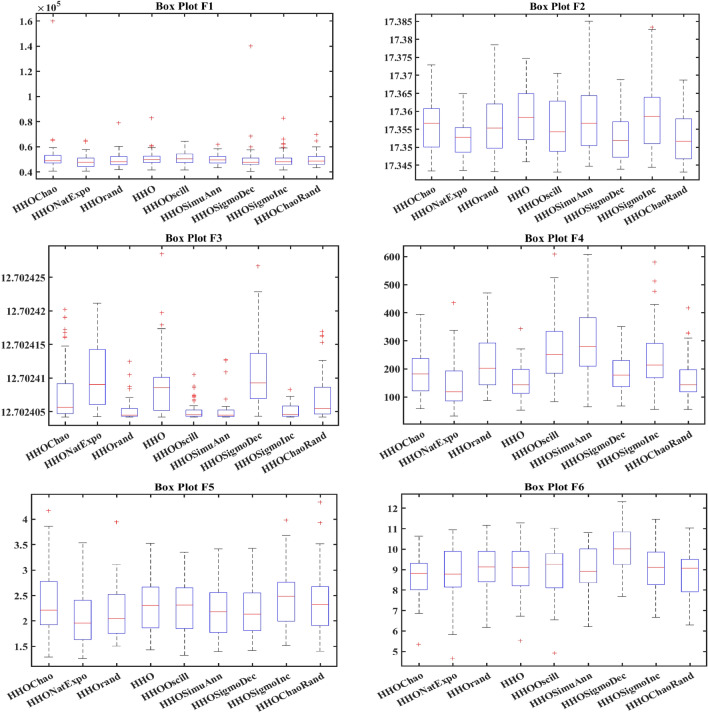

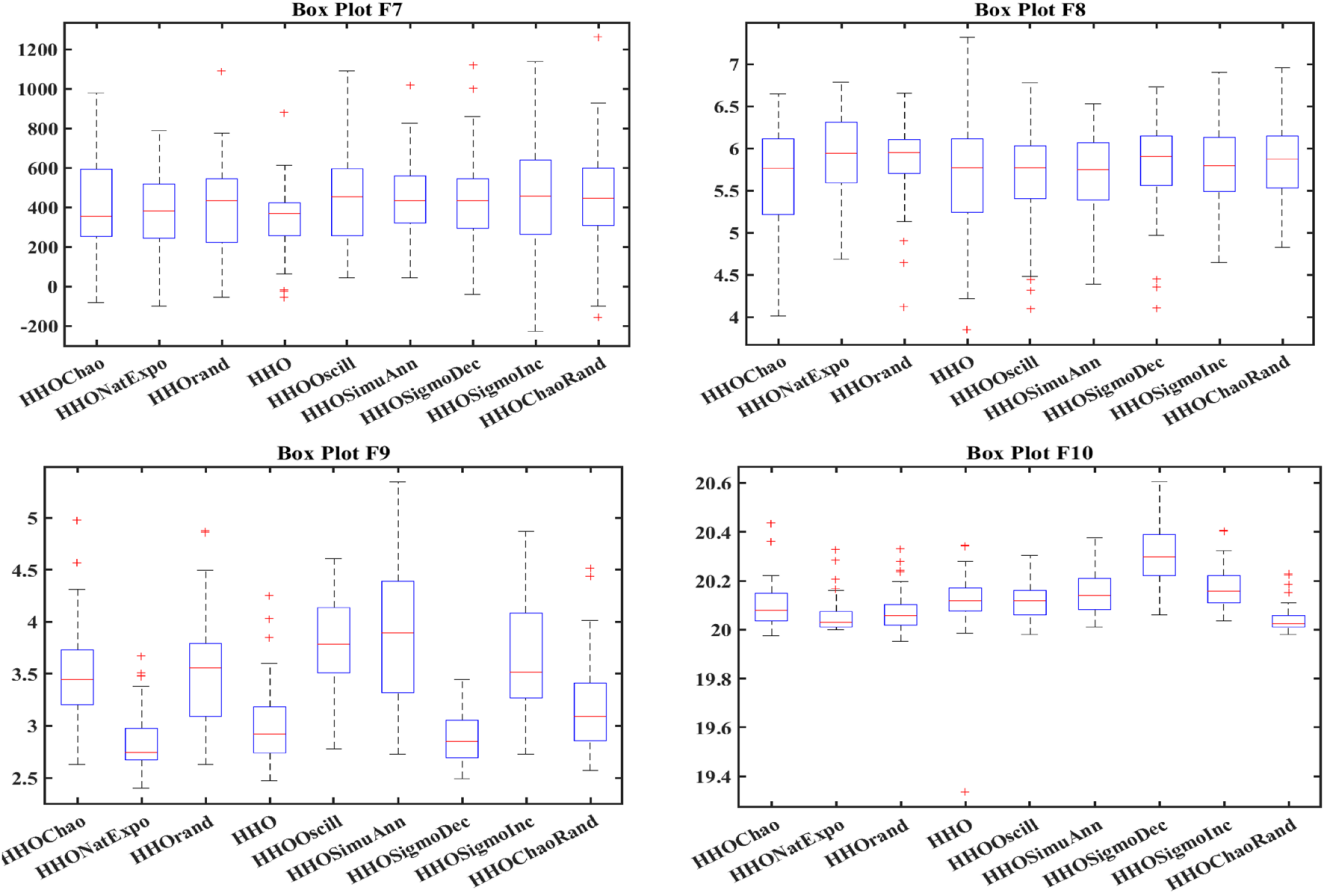
Box plots of mHHO with each operator for all benchmark functions (F1–F10).

From the overall f-rank value and average f-rank, it is evident that HHO-NatExpo is showing remarkable superiority over the other approaches and achieves the 1st rank followed by HHO-ChaoRand. In addition to the obtained simulation results, the convergence profiles of the mHHO algorithm using all operators are shown for a total of 500 iterations. The convergence characteristics for all benchmark functions (F1–F10) are also shown in Fig. 10. Interestingly it has been observed that the convergence profile of the HHO-ChaoRand and HHO-NatExpo approach is superior on all benchmark functions compared to other modified algorithms. Additionally, Fig. 11 displays a box plot analysis to further prove the performance of the algorithms on the benchmark functions. Based on the analysis of box plots, it is clear that the mHHO algorithm utilizing the Natural Exponent Operator (HHO-NatExpo) exhibits a lower median value and also a smaller interquartile range.

Also, from Table 4 it is clearly observed that the HHO_NatExpo secured the First rank. This result necessitates the selection of the HHO_NatExpo algorithm for comparison with the other MH algorithms for further designing of HRES. The outcomes presented in Table 5 show that the HHO_NatExpo can outperform other algorithms for functions F1, F2, F3, F4, F8, F9, and F10. In the sixth function, the EWOA algorithm gives the best possible outcomes in terms of mean and standard deviation values. For functions F5 and F7, the PSO algorithm shows a superior result as compared to the other algorithms. As a result, the HHO_NatExpo algorithm is shown to be successful for seven out of a total of ten numerical test functions, while the EWOA algorithm is effective for one function, and the PSO algorithm is effective for three functions. Overall, it has been determined that the HHO_NatExpo algorithm is the most successful algorithm for the CEC 2019 benchmark function.

**Table 5:** Comparison of simulation outcomes of HHO algorithm with different algorithms (CEC 2019 benchmark functions).

Function	Parameters	HHO-NatExpo	DE^60^	EWOA^61^	PSO^62^	Jde100^63^	NBA^64^	SCA^65^
F1	Mean	4.85E + 04	5.86E + 11	6.79E + 04	2.24E + 10	1.59E + 05	3.793E + 12	5.718E + 09
	std	5.34E + 03	8.36E + 11	1.25E + 04	1.73E + 10	1.60E + 05	3.242E + 12	7.133E + 09
	p-rank		–	–	–	–	–	–
	f-rank	1	6	2	5	3	7	4
F2	Mean	17.352	6.84E + 01	1.74E + 01	2.72E + 01	2.16E + 01	1.439E + 04	1.7451E + 01
	std	1.55E−03	1.42E + 02	4.2862E − 02	5.38E + 01	2.72E + 04	4.365E + 03	5.966E − 02
	p-rank		–	–	–	–	–	–
	f-rank	1	6	2	5	4	7	3
F3	Mean	12.702	1.27E + 01	1.27E + 01	1.27E + 01	1.31E + 06	1.271E + 01	1.270E + 01
	std	4.61E−06	1.900E − 03	0.00E + 00	7.5574E − 04	8.52E + 05	9.499E − 04	8.631E − 05
	p-rank		–	–	–	–	–	–
	f-rank	1	6	2	4	7	5	3
F4	Mean	1.42E + 02	1.54E + 03	1.56E + 02	1.20E + 03	3.48E + 05	2.436E + 04	1.082E + 03
	std	81.696	2.43E + 03	6.48E + 01	6.68E + 02	1.15E + 05	8.374E + 03	3.561E + 02
	p-rank		–	–	–	–	–	–
	f-rank	1	5	2	4	7	6	3
F5	Mean	2.093790767	2.12E + 00	8.13E + 00	1.65E + 00	1.67E + 05	6.964E + 00	2.170E + 00
	std	0.567547762	2.850E − 01	0.00E + 00	4.3138E − 01	8.43E + 04	1.745E + 00	1.252E − 01
	p-rank		–	–	+	–	–	–
	f-rank	2	3	6	1	7	5	4
F6	Mean	8.883	9.90E + 00	7.92E + 00	8.55E + 00	3.84E + 04	1.555E + 01	1.070E + 01
	std	1.242	1.47E + 00	1.29E + 00	1.15E + 00	2.06E + 03	1.286E + 00	6.285E − 01
	p-rank		–	+	+	–	–	–
	f-rank	3	4	1	2	7	6	5
F7	Mean	3.70E + 02	1.28E + 03	2.10E + 03	3.25E + 02	9.11E + 06	2.047E + 03	6.936E + 02
	std	2.16E + 02	4.22E + 02	0.00E + 00	2.89E + 02	4.38E + 08	3.248E + 02	1.585E + 02
	p-rank		–	–	+	–	–	–
	f-rank	2	4	6	1	7	5	3
F8	Mean	5.768	6.98E + 00	7.88E + 00	5.49E + 00	1.22E + 09	8.222E + 00	6.057E + 00
	std	0.493972	2.255E − 01	0.00E + 00	6.8809E − 01	4.39E + 08	4.517E − 01	4.714E − 01
	p-rank		–	–	–	–	–	–
	f-rank	1	3	5	4	7	6	2
F9	Mean	2.849	2.79E + 02	4.71E + 03	3.41E + 01	9.21E + 08	5.608E + 03	9.204E + 01
	std	0.304677	3.12E + 02	0.00E + 00	4.10E + 01	1.13E + 08	1.363E + 03	9.391E + 01
	p-rank		–	–	–	–	–	–
	f-rank	1	4	5	2	7	6	3
F10	Mean	20.05768623	2.04E + 01	2.09E + 01	2.00E + 01	1.54E + 06	2.108E + 01	2.044E + 01
	std	0.071915602	1.295E − 01	1.631E − 03	5.4341E − 02	7.46E + 05	1.630E − 01	8.691E − 02
	p-rank		–	–	–	–	–	–
	f-rank	1	4	5	2	7	6	3
W/L/T			0/10/0	01/09/0	03/07/0	0/10/0	0/10/0	0/10/0
Avg f-rank Value		1.4	4.5	3.6	30	6.3	5.9	3.3
Overall f-rank		1	5	4	2	7	6	3

#### Statistical testing

Two non-parametric tests Wilcoxon's rank-sum (p-rank) and Friedman rank (f-rank) test are performed to confirm the superior performance of the HHO_NatExpo algorithm. For analyzing the outcomes, wins (w), losses (l), and ties (t) are the first step in calculating the p-rank. The algorithm that wins against the HHO_NatExpo is assigned with a “ + ” sign to show its superior performance. Similarly, if the algorithms show worse outcomes as compared to the HHO_NatExpo algorithm, then assigned with the “−” sign. Finally “ = ” sign is assigned when there is no statistical difference between the compared algorithms. The HHO_NatExpo algorithm outperforms other MH algorithms in most of the functions and one can observe easily the result in the W/L/T row in Table 5. Additionally, the f-rank test assigns a distinct f-rank to every algorithm being evaluated and it is tabulated in the fourth row of every function in Table 5. The average f-rank is calculated for each algorithm and it is found that the proposed HHO_NatExpo algorithm achieved the Ist rank followed by the PSO and SCA algorithms.

### Results and discussion of HRES

The effectiveness and robustness of the proposed mHHO (HHO_NatExpo) algorithm on HRES is illustrated in this section since it is found to be optimal and the best one for designing reliable systems in comparison with other mHHO and MH algorithms. The simulation is performed with the population size set to 30 and the maximum iteration considered as 250. Furthermore, the technical and economic specifications for the simulation of HRES for components like Solar PV, Battery, DG, and others are presented in Table 6. The project's duration is taken as 20 years, while the interest rate is fixed at 3.5%.

**Table 6:** Technical specifications and associated expenses for various components of the HRES.

Component	Parameter	Values	Units
Solar PV	Rated power	1.00	kW
	Capital cost	469.03	$/kW
	Operation and maintenance cost	150	$/kW
	Derating factor	80	%
	Lifetime	20	Years
Wind (WT)	Rated power	1	kW
	Rated speed	10	m/s
	Cut out wind speed	25	m/s
	Cut in wind speed	2.5	m/s
	Wind turbine hub height	58	m
	Reference height	10	m
	Efficiency	96	%
	Capital cost	300	$/kW
	Replacement cost	300	$/kW
	Operation and maintenance cost	20	$/kW
	Lifetime	20	Years
Battery (BES)	Battery Capacity	100	kWh
	Battery depth of discharge	80	%
	Minimum state of charge (SOC_min_)	20	%
	Maximum state of charge (SOC_max_)	100	%
	Battery unit cost	190	$
	Replacement cost	150	$
	Operation and maintenance cost	0	$
	Efficiency	90	%
	Lifetime (year)	5	Years
Diesel generator (DG)	Capital cost	175	$/kW
	Replacement cost	175	$/kW
	Lifetime	10	Years
Inverter	Capital cost	40	$/kW
	Replacement cost	40	$/kW
	Operation and maintenance cost	10	$/kW
	Efficiency	96	%
Social	Fmax_DP	0.2	–
	Fmax_load	0.5	–
Other parameters	Real interest	3.5	%
	Project lifetime	20	Years

**Fig. 12:**
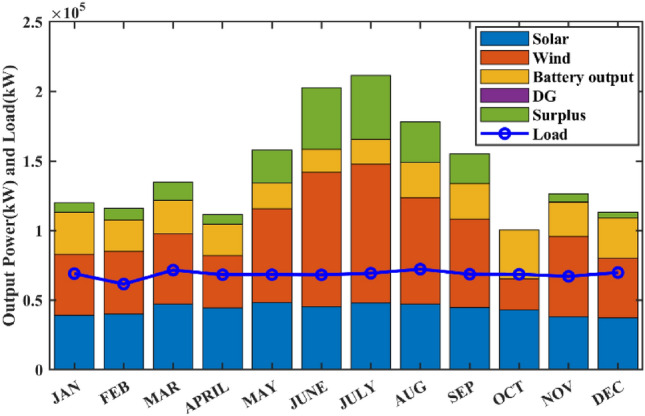
Output Power of all components and load demand.

The MATLAB 2021a program is used to simulate the experimental data. The simulation time step and duration considered are 1 h and 1 year respectively. The maximum ratings for solar PV panels, WTs, and number of batteries have been set at 300, 300, and 170 respectively for the purpose of comparing results. The optimum results includes the maximum rating of solar PV panels, WTs, the total quantity of batteries, and the highest possible rating for the DG. The viable and optimum solution is evaluated based on ASC, NPC, and COE. Table 7 displays the entire set of optimized results achieved for the case study using the proposed mHHO along with other MH algorithms such as basic HHO, LCA, MPO, AOA, EDO, NBA, and SCA algorithms. The findings proved that the mHHO algorithm yielded the lowest NPC and ASC, resulting in the least COE. The mHHO algorithm estimates the setup of a 299 kW solar PV system, 299 kW wind turbines, 169 batteries, and a 94 kW DG with an Annual System Cost (ASC) of $ 1,16,090 resulting in a COE of 0.1413$/kWh. From Table 7, it is observed that mHHO produces an 89% decrease in the value of COE against LCA, and also with basic HHO it offers 0.07%. Also, in comparison with other MH algorithms such as MPO, AOA, EDO, NBA, SCA, the proposed mHHO achieves around 0.49% decrease in the value of COE. This proved to be one of the most economical designs considering the ground realities present at the site location. The COE obtained using mHHO indicates that the proposed system can provide electricity to off-grid locations at a reasonable cost. Furthermore, Fig. 12 displays the monthly fluctuations in load demand along with the different components of power production of the HRES system over the course of a year. It can be seen from Fig. 12 that RES generates significant power during June and July making the period as one of the high-power production months. On the other hand, October is the least productive month in terms of power generation leading to increased utilization of battery power and DG.

**Table 7:** The optimal sizing results obtained using different algorithms.

Algorithm	Wind (kW)	Solar (kW)	Battery (units)	DG (kW)	Total annual cost ($/year)	NPC ($)	COE ($/kWh)
LCA	260.70	284.79	118.94	214.902	1.1082e + 06	1.5750e + 07	1.3485
MPO	300.00	300.00	170.00	94.2300	1.1612e + 05	1.6503e + 06	0.14200
AOA	300.000	300.00	170.000	94.2300	1.1612e + 05	1.6503e + 06	0.14200
EDO	300.00	300.00	169.56	94.2300	1.1611e + 05	1.6503e + 06	0.14200
NBA	300.00	300.00	170.00	94.2300	1.1612e + 05	1.6503e + 06	0.14200
SCA	299.68	300.00	170.00	94.2300	1.1611e + 05	1.6502e + 06	0.14200
HHO	300.00	299.76	170.00	94.2300	1.1610e + 05	1.6410e + 06	0.14131
mHHO	299.99	299.70	169.99	94.2300	1.1609e + 05	1.6499e + 06	0.14130

**Fig. 13:**
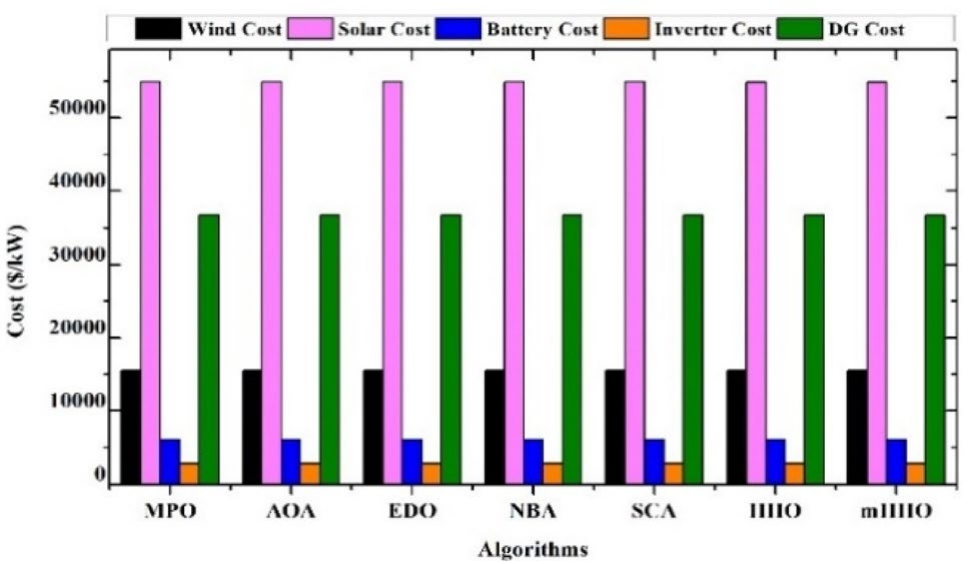
Cost of different components of HRES system using various algorithm.

Table 8 displays a comprehensive annualized cost evaluation of the hybrid system. The annualized costs of the system are determined using Capital Recovery Factor (CRF). Table 8 provides a thorough analysis of the cost of individual components and the annualized cost for the most efficient hybrid system. Yearly expenses are calculated using the CRF. Figure 13 demonstrates a comparative comparison of the cost of different components of HRES using various techniques. The results in Table 8 and Fig. 13 illustrates a significant decrease in the cost of several components when using mHHO leading to a reduction in the total annualized cost.

**Table 8:** Cost of each component of the proposed system by using different algorithms (PV-WT-BES-DG).

Algorithm	Wind cost ($/kW)	Solar cost ($/kW)	Battery cost ($/kW)	Inverter cost ($/kW)	DG cost ($/kW)	Total annual cost ($/year)
MPO	1.5515e + 04	5.4900e + 04	6.1262e + 03	2.8458e + 03	3.6729e + 04	1.1612e + 05
AOA	1.5515e + 04	5.4900e + 04	6.1262e + 03	2.8458e + 03	3.6729e + 04	1.1612e + 05
EDO	1.5515e + 04	5.4900e + 04	6.1104e + 03	2.8458e + 03	3.6742e + 04	1.1611e + 05
LCA	1.3483e + 04	5.2118e + 04	4.2864e + 03	2.8458e + 03	1.0355e + 06	1.1082e + 06
NBA	1.5515e + 04	5.4900e + 04	6.1262e + 03	2.8458e + 03	3.6729e + 04	1.1612e + 05
SCA	1.5498e + 04	5.4900e + 04	6.1262e + 03	2.8458e + 03	3.6739e + 04	1.1611e + 05
HHO	1.5516e + 04	5.4860e + 04	6.1270e + 03	2.8458e + 03	3.6743e + 04	1.1610e + 05
mHHO	1.5515e + 04	5.4857e + 04	6.1262e + 03	2.8458e + 03	3.6742e + 04	1.1609e + 05

**Fig. 14:**
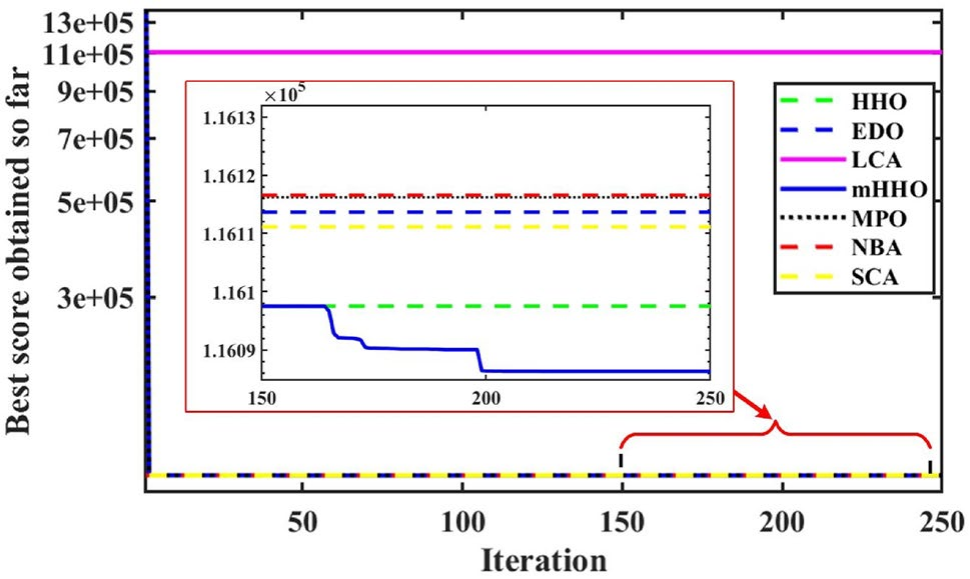
Comparison of different algorithm’s convergence rate.

Figure 14 shows an overview of the convergence characteristics of all algorithms. From an enhanced view of Fig. 14, it is observed that both the HHO and mHHO algorithm performs better compared to other MH algorithms but after the 165th iteration mHHO algorithm is giving better results than the basic HHO. Compared to the mHHO method, other algorithms required a longer time to simulate the proposed system. The ASC, cost of individual components, convergence curves, and sizing make the mHHO an imperative optimal algorithm for further HRES design.

**Fig. 15:**
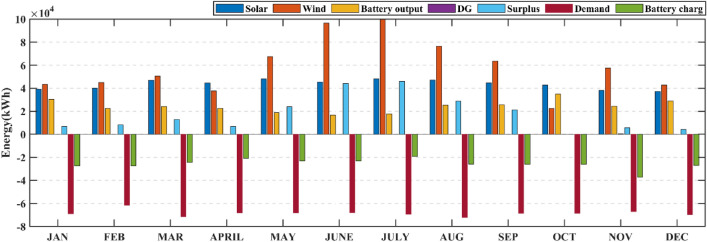
Assessment of monthly energy usage for the proposed case study.

It is observed that the proposed approach meets the whole energy requirement using solely solar, wind, and batteries except a infinitesimal duration using DGs. Figure 15 illustrates the monthly average energy balance and usage during a period of one year. It should be highlighted that the amount of energy produced by solar and wind has matched or is equal to the available resources (solar and wind). In October, when wind turbines are producing less power, a DG is used to meet the electricity need. Moreover it is revealed that during a given month, DG will be in operation whenever the cummulative energy produced by solar, wind and battery fails to satisfy the load demand. Throughout the remaining months in a year, a significant quantity of wind energy is produced because of the greater accessibility of natural resources. Except summer season, the utilization of battery banks is increased which means the power extracted from batteries is greater. It can be also seen from Fig. 15 that only in 3 months (June, July, and August), where more excess energy is available compared to other months of the year. Notably, wind energy makes up a significant portion of the contribution. Table 9 provides a concise comparison of the energy generated by every element of the designs derived from HHO, mHHO, LCA, EDO, MPO, NBA, and SCA. The total extra energy for the whole year utilizing mHHO is 209,100 kWh/year as shown in Table 9, which is lower than the results obtained using other techniques.

**Table 9:** Assessment of energy for various algorithms for the proposed system (PV-WT-BES-DG).

Algorithm	Wind (kWh)	Solar (kWh)	Battery in (kWh)	Battery out (kWh)	DG Energy (kWh)	Total demand served (kWh)	Excess energy (kWh/year)
MPO	7.0323e + 05	5.2256e + 05	3.0101e + 05	2.9230e + 05	436.2629	8.2182e + 05	2.0932e + 05
AOA	7.0323e + 05	5.2256e + 05	3.0101e + 05	2.9230e + 05	436.2629	8.2182e + 05	2.0932e + 05
EDO	7.0323e + 05	5.2256e + 05	3.0098e + 05	2.9226e + 05	466.5541	8.2182e + 05	2.0935e + 05
LCA	6.1112e + 05	4.9608e + 05	3.0427e + 05	2.9756e + 05	8925.50	8.2182e + 05	1.4114e + 05
NBA	7.0323e + 05	5.2256e + 05	3.0101e + 05	2.9230e + 05	436.2629	8.2182e + 05	2.0932e + 05
SCA	7.0248e + 05	5.2256e + 05	3.0108e + 05	2.9237e + 05	460.4861	8.2182e + 05	2.0886e + 05
HHO	7.0330e + 05	5.2306e + 05	3.0099e + 05	2.9228e + 05	467.1604	8.2182e + 05	2.0912e + 05
mHHO	7.0323e + 05	5.2215e + 05	3.0099e + 05	2.9228e + 05	467.2518	8.2182e + 05	2.0910e + 05

**Fig. 16:**
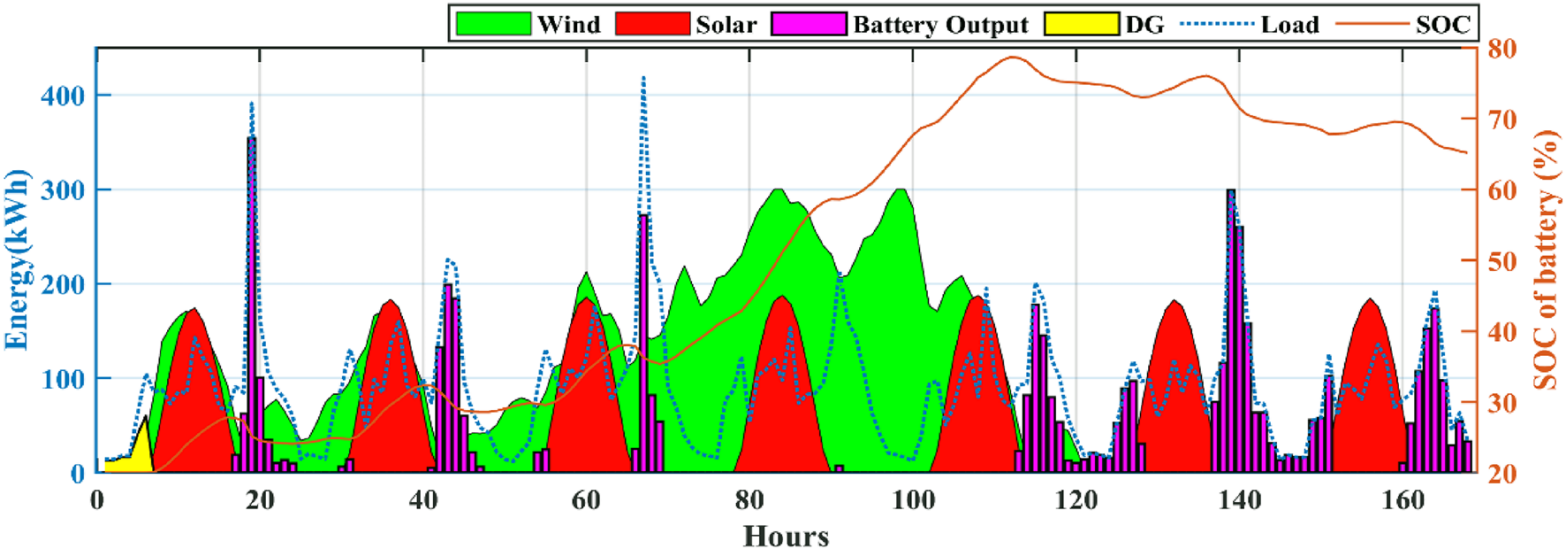
Energy balance with SOC for the 3rd week of October.

To better comprehend the power exchange among all the different elements of the system in a better way, a total of 2 weeks have been chosen. One week is taken in February when the load is lower whereas the second week is in October when the demand is greater. DG is used at these hours to supply electrical power to the system. Figure 16 displays a power exchange during 1 week in October. During this week DG is used when there is insufficient power from solar, and wind sources and also the battery's SOC is at or below the minimum threshold of 20% as outlined in the PMS. One can easily observe from Fig. 16 that, out of 168 h, the DG work for only 7 h in a week using mHHO. This has translated to 96% working of renewable sources with batteries by reducing the CO_2_ emissions to a bare minimum value.

**Fig. 17:**
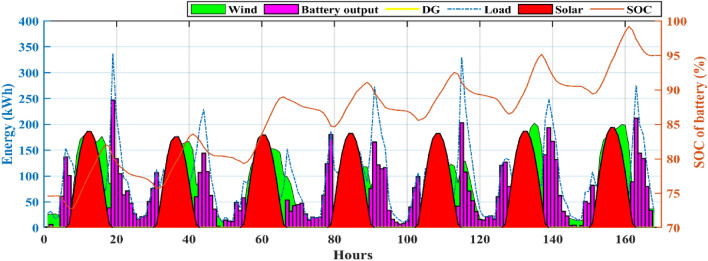
Energy balance and SOC of battery for the 1st week of February.

**Fig. 18:**
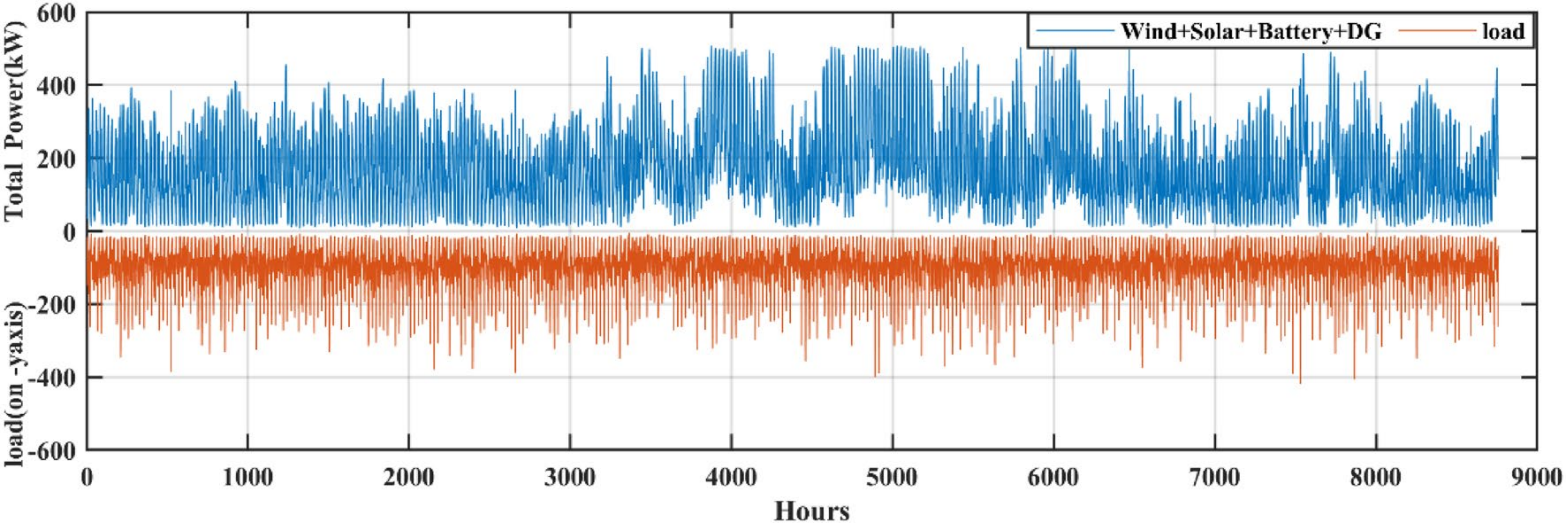
Total energy output and load.

Similarly, Fig. 17 illustrates the energy management during the last week of February. The data in Fig. 17 indicates that sufficient solar power and wind power are produced as a result of the abundant availability of both renewable sources. DG power is not required since the complete load requirement is satisfied only by batteries, solar, as well as wind energy sources. In addition to energy analysis, the total energy production and load characteristics for the complete year are displayed in Fig. 18. From Fig. 18, it is observed that the total output power (kW) surpasses the load (kW) showing that the total output power is enough to meet the load requirements.

**Fig. 19:**
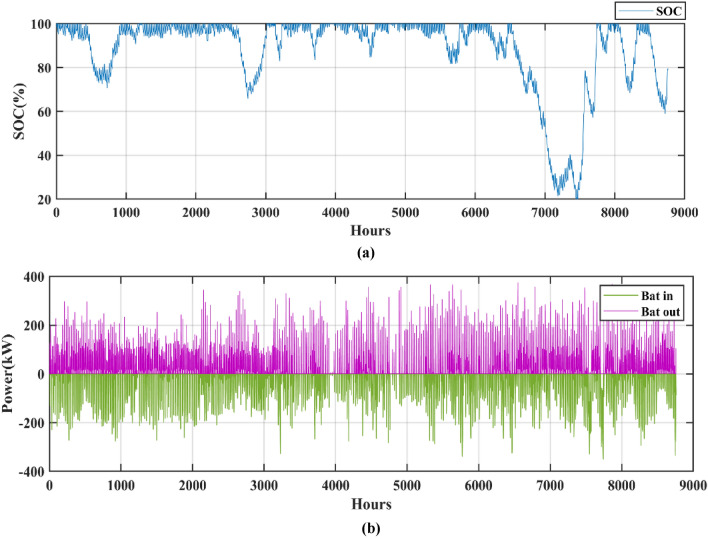
(**a**) State of charge of battery (%). (**b**) Total energy input and output of the battery during the period of 1 year.

Battery SOC evaluation is a crucial concern in systems that use batteries for storage. Figure 19a,b displays the fluctuations in the battery bank's SOC along with the input and output energy over the course of a year. The starting SOC and the lowest acceptable SOC are set at 100% and 20% respectively. Figure 19a shows that the battery SOC consistently stays within a predetermined limit. To understand better, it is considered that on January 1st, at 0000 h, the initial SOC of the battery banks is 100%. One can observe clearly from Fig. 19a that the SOC dropped to a minimum of 20% for only a short time during the whole year. Figure 19a demonstrates that the battery SOC is generally optimal with some exceptions during periods of limited natural resources. For example, during the month of January and high load demand in October–November, the SOC drops from its maximum value to a certain limit. This corroborates the result of minimal DG operation which is reported also in Fig. 16. If there is excess energy available above the charging rate and if SOC is also 100%, the excess energy may either be stored as a delayed load or dumped load. If the energy discharge exceeds the battery discharge rates, a DG will be used as the power generation source.

**Fig. 20:**
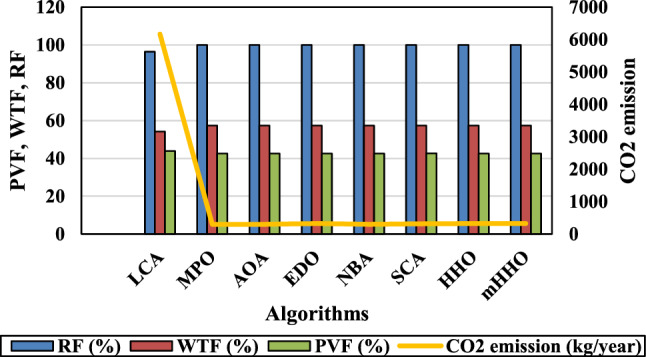
Comparison of different parameters.

**Fig. 21:**
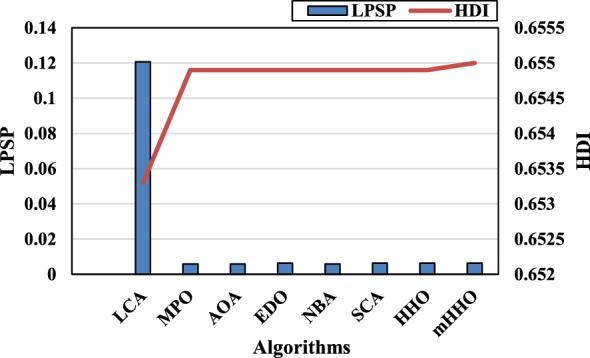
Comparison of LPSP and HDI for different algorithms.

Table 10 presents a comparative assessment of technical, social factors, and environmental factors such as LPSP, HDI, RF, WTF, PVF, and CO_2_ emissions using different MH algorithms for the proposed design. Based on the results, it is observed that mHHO outperforms other MH algorithms. Further, the comparative pictorial representation of these factors are displayed in Figs. 20 and 21. The CO_2_ emissions of the proposed HRES system have been reduced from 6165 kg/year using LCA to 322.7402 kg/year as reported by the mHHO method. The CO_2_ reduction is 94% which is a remarkable reduction. Also, in comparison with the basic HHO algorithm, the mHHO shows better results with a 0.9476% reduction in CO_2_ emission. The proposed mHHO also shows prominent results in HDI. It produces the HDI of 0.6550 which is almost advocated by WHO standards. From the above analysis and results, it is clearly proved that the mHHO is one the strong algorithm for implementation of HRES.

**Table 10:** Comparison of technical and social factors for the proposed system using several algorithms.

Algorithm	LPSP	HDI	RF (%)	WTF (%)	PVF (%)	CO_2_ emission (kg/year)
LCA	0.1207	0.6533	96.4353	54.1592	43.9637	6165.00
MPO	0.0059	0.6549	99.9469	57.3185	42.5929	301.3355
AOA	0.0059	0.6549	99.9469	57.3185	42.5929	301.3355
EDO	0.0063	0.6549	99.9432	57.3149	42.5903	322.2582
NBA	0.0059	0.6549	99.9469	57.3185	42.5929	301.3355
SCA	0.0062	0.6549	99.9440	57.2895	42.6169	318.0669
HHO	0.0063	0.6549	99.9430	57.3342	42.5708	322.7407
mHHO	0.0063	0.6550	99.9431	57.3340	42.5710	322.7402

## Conclusion

This work presented a new approach known as mHHO optimization algorithm for the 3E analysis of HRES. Primarily the paper is focussed on Energy assessment, Economic evolution (COE, NPC, and ASC), and Environmental analysis (PVF, WTF, RF, CO_2_ emission). In addition, the technical and social factors such as LPSP and HDI for HRES (PV-Wind-Battery-DG) are also evaluated. To obtain a practical HRES, mHHO optimization algorithm is utilized to achieve the optimal system design and to determine the appropriate sizes of HRES components. The proposed mHHO algorithm is modified using 9 different operators and tested on CEC 2019 benchmark test functions. The comparative analysis is carried out based on statistical tests such as the f-rank test with convergence and box plot studies. Then, the algorithm that has yielded a higher rank i.e. mHHO (HHO-NatExpo) which has good performance and a good convergence profile compared to others is selected for the HRES problem solving in the chosen case study location. The proposed mHHO algorithm demonstrated superior performance by achieving the lowest COE of 0.14130$/kWh, ASC of 116,090$/year, and NPC of 1,649,900$. Furthermore, mHHO has exceptional efficacy in reducing the environmental pollutant CO_2_ emission of which is accounted to a mere 322.7402 kg/year while maintaining HDI at the prescribed level. From an optimization perspective, it has been demonstrated that the mHHO algorithm has superior outcomes in 3E assessments compared to basic HHO and other MH-algorithms (LCA, MPO, AOA, EDO, NBA, and SCA algorithm). The results of the simulation indicated that using renewable resource might potentially serve as high reliability for optimizing hybrid energy systems. The incorporation of RES into the hybrid system has been seen to provide the advantage of meeting the entire energy demand by reaching 100% fulfillment in the near future. On the other hand, one of the crucial problems to address is the examination of the mHHO algorithm's behavior throughout the exploration and exploitation phases. Due to the algorithm's stochastic nature, there is a significant probability that it can diverge from the global optimal point and get stuck in local optima thereby reducing its reliability for different optimization challenges. In addition to said limitations, further the incorporation of adaptive parameters does not consistently provide a well-balanced functioning and may sometimes lead to premature convergence.

Based on the findings and implementation challenges, the following recommendations are suggested for future work enhancement and development. A laboratory-scale experimental setup can be developed to facilitate deeper research into the performance of HRES in harsh environments. The simulation model may also be created to understand the HES's power flow regulation process. Consequently, this work helps to raise potential awareness about the installation of RESs in different parts of the rural areas intending to facilitate an economically sustainable energy supply.

## Data Availability

The datasets used and/or analysed during the current study available from the corresponding author on reasonable request.
